# The Biomechanical Mechanism of Cranial Implant Exposure Following Decreased Intracranial Pressure: A Finite Element Analysis Study

**DOI:** 10.3390/biomedicines14102348

**Published:** 2026-10-15

**Authors:** Wentao Huang, Ruizhe Zheng, Linfeng Zhang, Dongchang Shao, Jiayu Chen, Lijian Lang, Wenqian Shi, Jiuqi Yan, Zengxin Qi, Xuehai Wu

**Affiliations:** 1 Department of Neurosurgery, Huashan Hospital, Shanghai Medical College, Fudan University, Shanghai 200040, China; hhuangwt7777@163.com (W.H.); ruizhedoctor@126.com (R.Z.); ironman1995@163.com (L.Z.); dcshao24@m.fudan.edu.cn (D.S.); 23111220059@m.fudan.edu.cn (J.C.); lang_lijian@163.com (L.L.); 15653762356@163.com (W.S.); yanjiuqi0610@163.com (J.Y.); 2 National Center for Neurological Disorders, Shanghai 200040, China; 3 Shanghai Key Laboratory of Brain Function and Restoration and Neural Regeneration, Shanghai 200040, China; 4 Neurosurgical Institute of Fudan University, Shanghai 200040, China; 5 Shanghai Clinical Medical Center of Neurosurgery, Shanghai 200040, China; 6 State Key Laboratory of Medical Neurobiology and Ministry of Education (MOE) Frontiers Center for Brain Science, School of Basic Medical Sciences and Institutes of Brain Science, Fudan University, Shanghai 200032, China; 7 Department of Neurosurgery, Third Division General Hospital, Xinjiang Production and Construction Corps, Tumushuke 843900, China

**Keywords:** cranioplasty, intracranial pressure, titanium mesh exposure, polyetheretherketone

## Abstract

**Background**: Skull prosthesis is widely used for cranioplasty, yet long-term implant exposure remains a challenging complication frequently requiring reoperation. Clinical observations suggest an association between low intracranial pressure (ICP) and progressive scalp thinning, with possible progression to mesh erosion and implant exposure. However, the potential biomechanical mechanism by which dynamic ICP fluctuations may contribute to scalp atrophy and implant exposure has not been characterized owing to limitations of in vivo measurement. **Methods**: To investigate the potential biomechanical basis of low ICP-induced exposure, finite element analysis was used to simulate the mechanical responses of three cranial implant designs (combine-pattern titanium mesh, circular-pore titanium mesh, polyetheretherketoneplate) and overlying scalp under dynamic ICP. A skull model was generated from Computed Tomography data. Loading included a uniform 50 N force with continuously graded ICP (−15 to 30 mmHg) and localized 50 N forces at central and off-central regions under discrete ICP levels. **Results**: This study provides the biomechanical insights that low ICP may amplify stress on scalp overlying combined-pattern titanium mesh, supporting the “low ICP–scalp atrophy–exposure” cascade. The circular-pore titanium mesh consistently exhibited low and stable mechanical responses across all pressures, whereas polyetheretherketone plate demonstrated nonlinear behavior with aberrantly high-stress under specific boundary conditions. The central implant region was identified as a potentially vulnerable zone. **Conclusions**: Low ICP significantly elevates central scalp stress, suggesting it may be a mechanical contributor to implant exposure. Circular-pore titanium mesh and polyetheretherketone plate showed more favorable biomechanical behavior, warranting further validation. In high-risk scenarios, central porosity reduction or flexible transitions may be considered potential design modifications to mitigate exposure.

## 1. Introduction

In recent years, titanium mesh has emerged as the predominant implant material for the surgical reconstruction of cranial defects in neurosurgical practice. This preeminence is largely attributable to its favorable intraoperative adaptability, robust fixation stability, and capacity for satisfactory immediate restoration of cranial contour. Nevertheless, long-term complications associated with titanium mesh implantation—particularly the progressive attenuation of the overlying scalp culminating in implant extrusion—have increasingly been recognized as a critical impediment to achieving optimal long-term therapeutic outcomes [1]. Titanium mesh is associated with a statistically significantly greater risk of exposure than polyetheretherketone plate (PEEK), yet the clinical application of PEEK is limited by its elevated cost [2,3,4]. In a retrospective analysis of 401 patients, Kwiecien et al. particularly noted that titanium mesh is prone to cause progressive thinning/atrophy of the overlying scalp flap, especially in high-risk patients with composite soft-tissue defects; this tendency appeared more pronounced when the mesh was placed directly over the dura, whereas placement over bone did not seem to cause a similar effect [5]. According to aggregated clinical data, the reported incidence of titanium mesh exposure ranges from 4.6% to 17%, and a subset of affected patients ultimately necessitates secondary surgical intervention for implant removal [5,6,7,8,9]. Consistent with the principles discussed by Zanotti et al., titanium mesh exposure may be more likely when the overlying soft tissue is thin, poorly vascularized, or subjected to tension/pressure, and delayed presentation beyond 6 months should be anticipated [10]. Prior literature has delineated several independent predictors of exposure, with advanced age, diabetes, and pronounced bone flap concavity conferring a substantially augmented risk [1,10,11].

Moreover, emerging clinical evidence has increasingly implicated postoperative intracranial hypotension as a central pathogenic driver in the evolution of titanium mesh exposure. Notably, Zheng et al. documented that among patients presenting with mesh exposure and a concomitant history of ventriculoperitoneal shunt placement, low-pressure hydrocephalus was identified in 90% of the affected cohort [12]. Maqbool et al., in a retrospective radiographic review, identified the presence of epidural dead space in a subset of exposure cases; although this finding did not attain statistical significance [7], it nonetheless suggests that aberrant negative pressure gradients may exist in certain patients presenting with titanium mesh exposure. Pathological investigations have further demonstrated that at sites of mesh exposure, epithelial tissue can be observed migrating along the dural surface, characterized by the presence of dermal appendage structures. This phenomenon is interpreted as the seeding and subsequent proliferation of epidermal tissue beneath the titanium mesh under the influence of negative pressure suction [10,13,14]. In summary, the “internal suction effect” engendered by intracranial hypotension drives the process of titanium mesh exposure through two synergistic mechanisms.

Despite these clinical observations, the underlying biomechanical mechanisms by which decreasing intracranial pressure (ICP) precipitates scalp thinning and titanium mesh exposure remain entirely unexplored and speculative, largely owing to the technical challenges associated with conducting mechanical studies on physical models capable of dynamic ICP adjustment. Moreover, the varying textures/patterns of cranioplasty implants also influence their mechanical performance [15,16], making computational simulation essential in this context. Therefore, the objective of this study was to employ finite element analysis (FEA) to simulate the dynamic mechanical alterations occurring in cranioplasty materials and the overlying scalp under varying ICP conditions, thereby providing biomechanical evidence to inform clinical practice and the development of improved implant designs.

## 2. Materials and Methods

**Figure 1:**
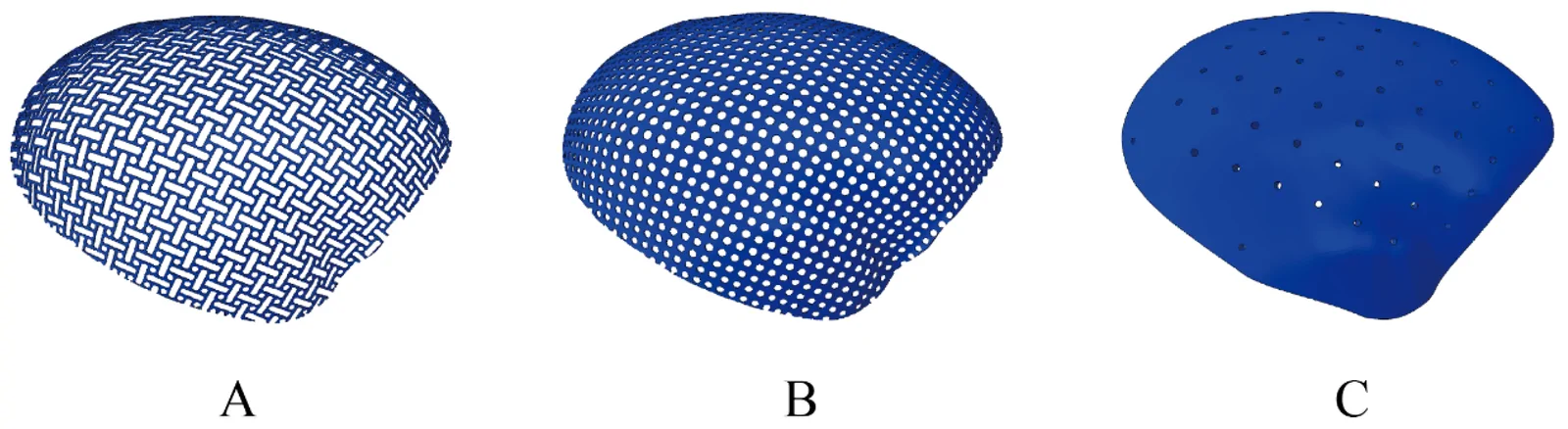
Morphological characteristics of the three cranioplasty materials. (**A**) Combine-pattern titanium mesh; (**B**) circular-pore titanium mesh; (**C**) PEEK: polyetheretherketone plate.

In this study, the biomechanical responses of cranial reconstruction assemblies utilizing three distinct cranioplasty materials: composite-pattern titanium mesh(Combine), circular-pore titanium mesh(Circle), and PEEK; Figure 1 were analyzed under four distinct mechanical loading scenarios.

### 2.1. Experimental Subjects

This observational study was approved by the Ethics Committee of Huashan Hospital, Fudan University (KY2024-1323), approved on 12 November 2024. A three-dimensional geometric model representing the authentic anatomical structure of a human skull was generated using Computed Tomography (CT) images obtained from a 56-year-old female patient. The CT scan was performed at Huashan Hospital using a TOSHIBA Aquilion ONE scanner (device serial number SERIALNO, Toshiba Medical Systems Corporation, Otawara, Tochigi, Japan) with a slice thickness of 0.6 mm.

### 2.2. Construction of the Cranial 3D (Three-Dimensional) Finite Element Model

The CT images were utilized for three-dimensional reconstruction of the skeletal structures. CT scan data were imported into Materialise Interactive Medical Image Control System (MIMICS) modeling software (version 21.0 Materialise NV, Leuven, Belgium) for automated generation of the initial skull model. Two neurosurgeons performed manual segmentation of the skull to ensure that the anatomical structure conformed to the classic question-mark incision utilized in decompressive hemicraniectomy. The processed data were saved in Standard Tessellation Language/Stereolithography (STL) format. Subsequently, the STL file was directly imported into Automatic Net Generation for Structural Analysis (ANSA) software (version 2021 BETA CAE Systems SA, Thessaloniki, Greece) for model processing and editing, encompassing surface repair and smoothing to yield a geometrically continuous and high-fidelity computational model. Assembly and structural optimization of the processed model were finalized within ANSA, followed by mesh generation. Mesh quality control metrics were as follows: aspect ratio ≤ 5; minimum element height ≥ 0.2; and element characteristic length ranging from 0.2 to 2. Internal angles for quadrilateral elements ranged from 30° to 150°, and for triangular elements from 20° to 140°. Local mesh refinement was applied to the implant and adjacent skull regions, with an element size of 0.4 mm in these areas and 1.5 mm in the remaining regions. Mesh convergence was verified through sensitivity analysis to ensure stable results. Solid components were modeled using Abaqus first-order tetrahedral elements (C3D4, 4-node linear tetrahedron), while shell structures were modeled using S3 (3-node linear triangular shell) elements. The Combine, Circle, and PEEK models consisted of 1,304,898, 1,425,332, and 1,519,269 elements, respectively.

A region of the skull was removed from the model to simulate a patient who had undergone a decompressive hemicraniectomy, as described by Becker et al. [17]. Three anatomically contoured cranioplasty implant models, referencing the contralateral intact skull, were constructed on this hemicraniectomy model. Model 1 represented a composite with a thickness of 0.6 mm, inner circle radius of 1.175 mm, outer circle radius of 1.8 mm, solid strut width of 0.625 mm, and oblong perforations with a length-to-width ratio of 3:1, secured to the skull with 12 screws. Model 2 simulated a uniformly distributed large-pore circular titanium plate with a thickness of 0.6 mm, screw-hole diameter of 2.67 mm, and center-to-center hole spacing of 4.19 mm, secured with 12 screws. Model 3 represented a uniformly thick PEEK plate of 4 mm thickness, with circular holes of 2 mm diameter spaced 12 mm center-to-center, secured to the skull using 5 connecting plates, each affixed with two screws. Multi-point constraint (MPC) coupling was applied between the superior aspect of the screws and the implant holes to simulate bolted connections. Similarly, MPC coupling was applied between the inferior aspect of the screws and the skull to simulate screw engagement within the bone.

### 2.3. Material Properties

The material properties of the skull and scalp were adopted from previously published head finite element models [18,19,20,21]. For the titanium mesh, screws, and PEEK implant, the elastic constants were obtained from manufacturers’ technical data for the corresponding commercial products and cross-checked against values reported in the literature. The elastic modulus and Poisson’s ratio were defined as follows: skull (E = 15,000 MPa, ν = 0.26); titanium mesh (E = 110,000 MPa, ν = 0.3) [22]; screws (E = 110,000 MPa, ν = 0.3) [22]; scalp (E = 16.8 MPa, ν = 0.45) [23]; and PEEK (E = 4000 MPa, ν = 0.40) [22,24].

### 2.4. Measurement Methods

Two distinct external force application modes were applied to the three models.

Mode 1: Prior investigations have frequently employed a localized application of a 50 N force to the central region of the implant [25]. However, such concentrated loading inadequately simulates the physiological scenario in which forces are distributed across the entire cranioplasty region, such as when resting the head on a pillow. Therefore, this study introduced a novel loading paradigm in which a uniformly distributed normal pressure load, equivalent to a total external force of 50 N, was applied over the scalp surface covering the cranial implant. The loaded area was defined by projecting the implant footprint onto the outer scalp surface. The pressure magnitude was calculated as 50 N divided by this loaded area and was applied normal to the local scalp surface. Concurrently, ICP was dynamically adjusted to simulate and compute the alterations in stress and strain experienced by the scalp and cranial implant under conditions of decreasing or increasing ICP. ICP was applied as a distributed surface pressure on the inner surface of the skull, the undersurface of the implant, and the inner surface of the scalp exposed through the implant pores, rather than as a fixed-direction traction. The pressure direction was determined by the normal of the loaded surface, and its continuous variation from −15 to 30 mmHg was controlled by an amplitude curve. Internal loading consisted of a continuously graded range of ICP from −15 to 30 mmHg, applied in increments of 0.735 mmHg (equivalent to 1 cm H_2_O). ICP values from −15 to 5 mmHg were classified as the low ICP group, 5–15 mmHg as the normal ICP group, and 15–30 mmHg as the high ICP group.

**Figure 2:**
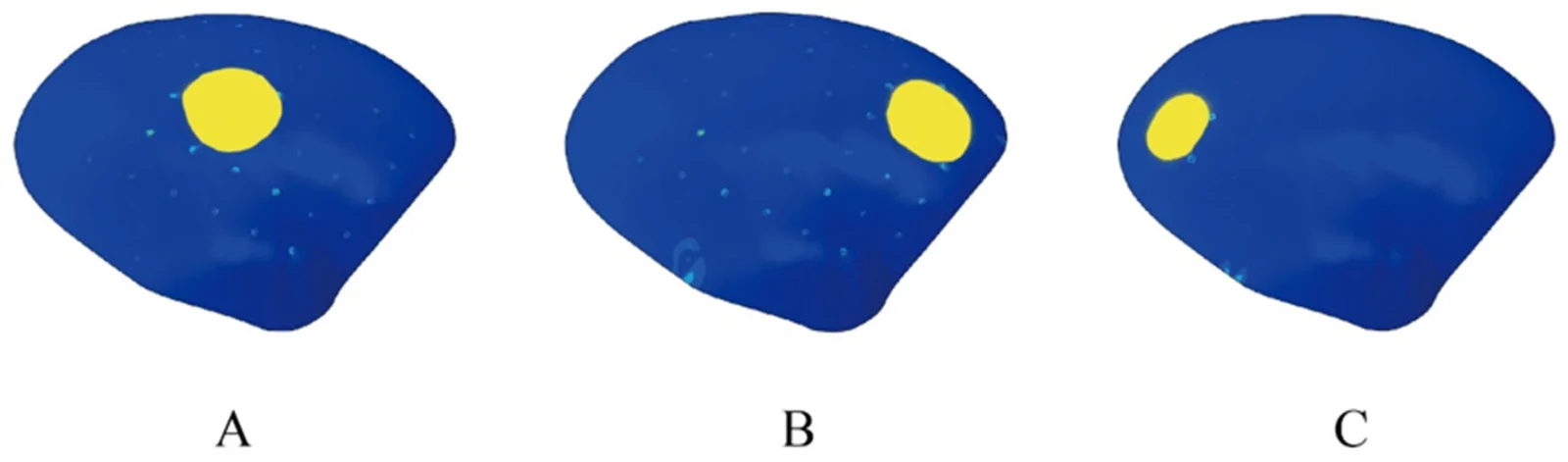
This figure illustrates the three locations where the external force is applied in Mode 2. The yellow-colored regions indicate the positions of the 50 N force application in loading mode 2, with (**A**) at the center, and (**B**) and (**C**) on the right and left sides of the central area, respectively.

Mode 2: Consistent with methodologies described in prior literature [18,25], the load was not applied as a concentrated force. Instead, a total external force of 50 N was applied as a uniformly distributed surface pressure over a circular scalp area of approximately 2 cm in diameter, directed normal to the local scalp surface overlying the cranial implant. The location of this external force application was varied: at the central region of the implant (Position A) and at regions to the left (Position C) and right (Position B) of the center. ICP was sequentially set at −5, 10, and 25 mmHg in graded fixed increments of 15 mmHg. Figure 2 shows the location where the external force is applied.

**Figure 3:**
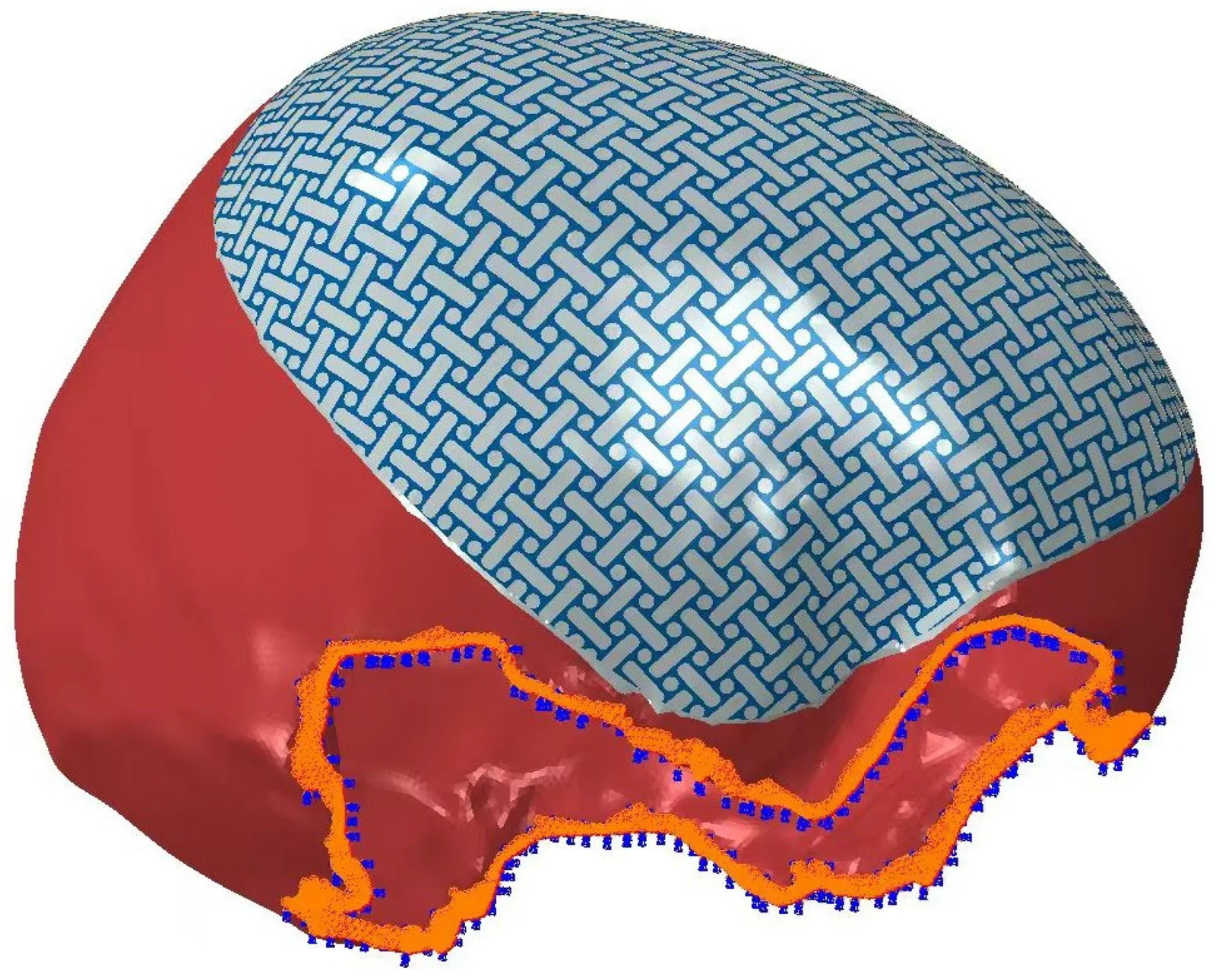
Schematic illustration of the fixed boundary constraint applied to the skull model. Identical fixed boundary constraints were applied to the truncated inferior surface of the skull, restricting all six degrees of freedom (three translational and three rotational). The blue region indicates the fixed constraint.

For steady-state intracranial-extracranial pressure analyses, identical contact pairs were defined across the three models: contact interfaces between the undersurface of the implant and the outer table of the skull were assigned a friction coefficient of 0.1. Identical fixed boundary constraints were applied to the truncated inferior surface of the skull, restricting all six degrees of freedom (three translational and three rotational). Figure 3 shows the fixed boundary constraint.

Global maximum stress and displacement values were recorded for the scalp (scalp stress, scalp displacement) and for the implant material (implant stress, implant displacement) across all models. These maxima were compared to evaluate the biomechanical loading conditions experienced by the scalp and the implant. Stress calculations were performed using the implicit analysis standard module of Abaqus software (version 6.13 Dassault Systèmes Simulia Corp., Providence, RI, USA).

## 3. Results

This study employed finite element simulation under two distinct external loading modes to analyze the biomechanical responses of three cranial implant materials—Combine, Circle, and PEEK—and the overlying scalp across a continuously graded ICP range of −15 to 30 mmHg under uniform loading, as well as under three discrete loading conditions (Positions A, B, C). The results revealed significant disparities in the mechanical responses among the three materials and their corresponding overlying scalp. The Combine exhibited the highest stress and displacement values, the circular-pore mesh demonstrated the lowest and most stable responses, and the PEEK implant displayed nonlinear behavioral characteristics.

### 3.1. Uniform External Load Distribution Condition

#### 3.1.1. Combine-Pattern Titanium Mesh (Combine)

**Figure 4:**
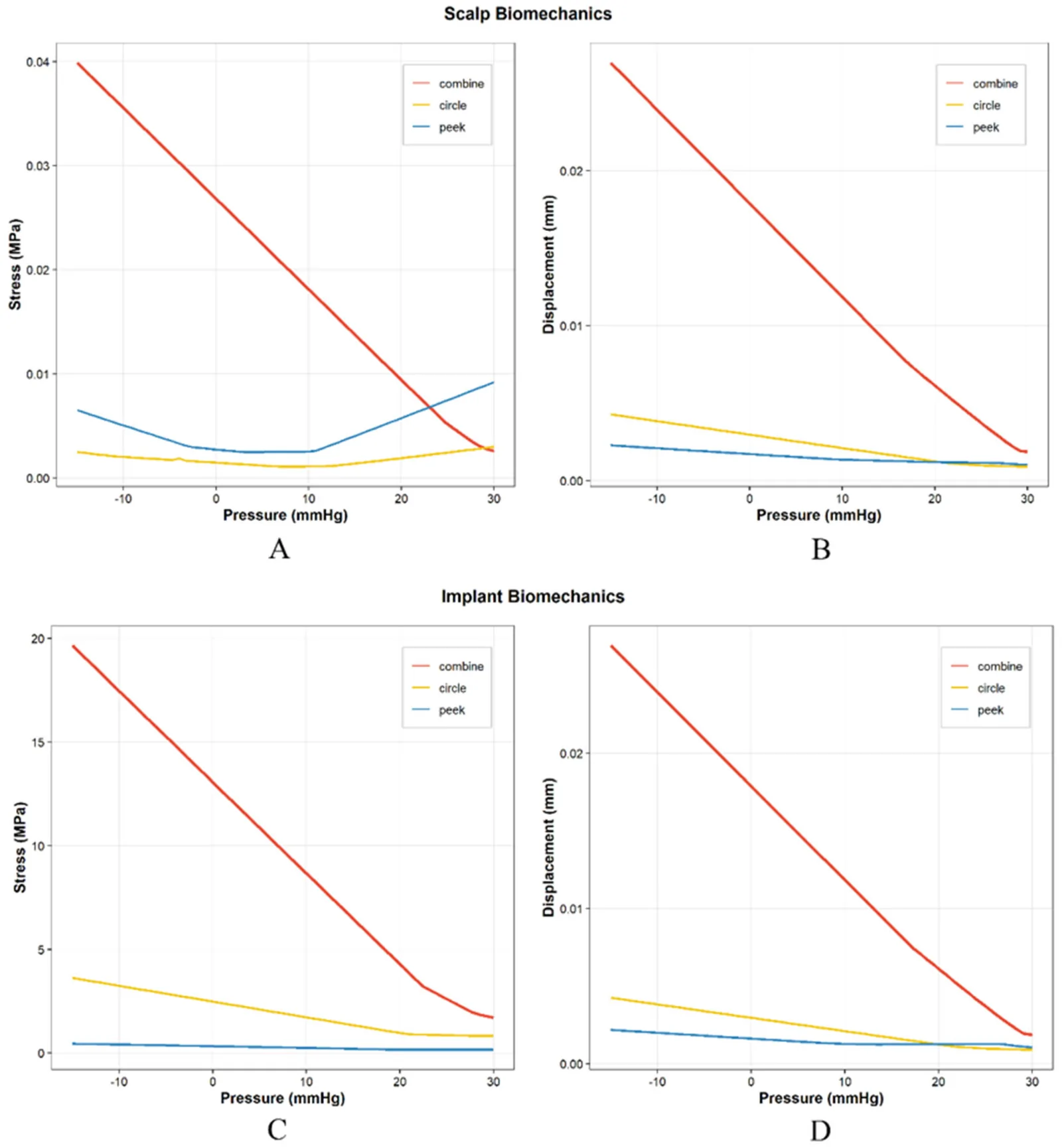
Variation in the maximum scalp stress, maximum scalp displacement, maximum implant stress, and maximum implant displacement as a function of intracranial pressure under Mode 1 (uniform external loading). (**A**) Maximum scalp stress for the three materials. (**B**) Maximum scalp displacement for the three materials. (**C**) Maximum implant stress for the three cranioplasty materials. (**D**) Maximum implant displacement for the three cranioplasty materials. Combine: Combine-pattern titanium mesh; Circle: circular-pore titanium mesh; PEEK: polyetheretherketone plate.

Stress trend: Both scalp stress and implant stress exhibited a steep decline with increasing ICP. At the extreme negative pressure of –15 mmHg, the scalp stress reached its global maximum of 0.0399 MPa, while the implant interior experienced a high stress of 19.66 MPa. As ICP rose into the positive range and reached 30 mmHg, the scalp stress decreased to 0.0026 MPa, and the implant stress dropped sharply to 1.71 MPa (Figure 4).

Displacement trend: At an ICP of –15 mmHg, both the scalp displacement and implant displacement attained their peak values, which were 0.02696 mm and 0.02698 mm, respectively. Thereafter, with increasing ICP, the displacement displayed a nearly linear and steep downward trend. When the ICP increased to 30 mmHg, the scalp displacement decreased to 0.00187 mm, and the implant displacement decreased to 0.00187 mm (Figure 4).

#### 3.1.2. Circular-Pore Titanium Mesh (Circle)

Stress trend: The scalp stress was 0.00249 MPa at –15 mmHg. As ICP increased, the stress exhibited an initial decrease followed by a subsequent rise, reaching a global minimum of 0.001083 MPa at 7.05 mmHg. After passing this nadir, the scalp stress underwent a slight increase, rising to 0.00299 MPa at 30 mmHg. The implant stress declined steadily with increasing ICP, from 3.63 MPa at –15 mmHg to 0.83 MPa at 30 mmHg (Figure 4).

Displacement trend: In the Circle group, both the scalp and implant displacements remained within a low range under negative and positive ICP. At –15 mmHg, the scalp displacement was 0.00428 mm and the implant displacement was 0.00426 mm; as ICP rose to 30 mmHg, these values decreased to 0.00090 mm and 0.00090 mm, respectively (Figure 4).

#### 3.1.3. PEEK Implant

Stress trend: The scalp stress of PEEK exhibited an “initial decrease followed by a subsequent rise” pattern. At –15 mmHg, the stress was 0.00651 MPa; it gradually declined with increasing ICP and reached a global minimum of 0.002482 MPa at 3.375 mmHg. After passing this nadir, the scalp stress displayed a pronounced monotonic increase, attaining a global maximum of 0.0092 MPa at 30 mmHg. The implant stress remained at an extremely low level throughout, declining slightly from 0.45 MPa at –15 mmHg to 0.16 MPa at 30 mmHg (Figure 4).

Displacement trend: The PEEK material exhibited the smallest displacement among the three materials. At –15 mmHg, the scalp displacement was 0.00228 mm and the implant displacement was 0.00219 mm; as ICP increased to 30 mmHg, the scalp displacement decreased to 0.00105 mm, and the implant displacement decreased to 0.00107 mm (Figure 4).

#### 3.1.4. Stress Crossover Between Materials in Scalp Stress

A key mechanical crossover point exists between PEEK and Combine materials in terms of scalp stress (Figure 4A). As the scalp stress of Combine decreases sharply with increasing ICP, while that of PEEK exhibits a reverse upward trend after passing its nadir (3.375 mmHg), the two curves intersect. Quantitative data indicate that the scalp stress of PEEK first exceeds that of Combine within the ICP range of 23.22 mmHg to 23.955 mmHg. Specifically, at 23.955 mmHg, the scalp stress of the PEEK group (0.007116 MPa) was already higher than that of the Combine group (0.006016 MPa). At 30 mmHg, this discrepancy further widened, with the PEEK group’s scalp stress (0.009206 MPa) being markedly higher than that of the Combine group (0.002595 MPa). It should be noted, however, that although the scalp stress under intracranial hypertension was relatively higher in PEEK, it remained substantially lower than the stress levels observed under intracranial hypotension (Figure 4). Detailed data are provided in the Supplementary Materials (Tables S1–S9).

#### 3.1.5. Correlation Analysis

##### Implant Stress and Displacement

For all three materials, the maximum implant stress and maximum implant displacement were very strongly negatively correlated with ICP (|Correlation coefficient (r)| > 0.93, all *p* < 0.0001). All *p* values were below 0.0001, indicating highly significant correlations and very high reliability of the results (Table 1).

##### Scalp Displacement

The maximum scalp displacement of the three materials was also very strongly negatively correlated with ICP (|r| > 0.97, all *p* < 0.0001), with the correlation approaching a linear dependence: increasing ICP led to a pronounced decrease in scalp deformation (Table 1).

##### Turning-Point Phenomenon in Scalp Stress

The maximum scalp stresses of the Circle and PEEK materials exhibited non-monotonic trends; however, the segmental correlations before and after the respective turning points were both extremely significant (|r| > 0.97, *p * < 0.0001). For Circle, the correlation direction was completely reversed around the turning point (ICP = 7.05 mmHg): before the turning point, there was a very strong negative correlation (r = –0.9923, *p* < 0.0001), whereas after the turning point, there was a very strong positive correlation (r = 0.9891, *p* < 0.0001); the absolute values of the correlation coefficients in both segments exceeded 0.98, underscoring the statistical significance. For PEEK, the turning point was located at 3.375 mmHg, with a very strong negative correlation before the turning point (r = –0.9824, *p* < 0.0001) and a very strong positive correlation after the turning point (r = 0.9823, *p* < 0.0001); the correlation strength was similarly high and the *p* values were extremely small, indicating that the reversed trend is highly reliable. Detailed data are provided in Table 1.

Using the Circle model as a reference, multiplicative factors were calculated for the comparisons of Combine vs. Circle, Combine vs. PEEK, and PEEK vs. Circle within the uniform loading ICP range of −15 to 15 mmHg (Table 2). Under identical loading conditions, scalp stress for the Combine model was 20–22 times greater than that for the Circle model, and implant stress was 6–7 times greater. Compared to PEEK, scalp stress for the Combine model was 3–10 times higher, and implant stress was 37–98 times higher. Under normal pressure ranges, implant stress, implant displacement, and scalp displacement for PEEK were lower than those for Circle, whereas scalp stress for PEEK and Circle was comparable. However, under high positive pressure (>20 mmHg), scalp stress increased for PEEK while remaining minimal for Circle.

This table presents the differences in stress and displacement among the materials. As shown, the results differ by orders of magnitude. Combine: Combine-pattern titanium mesh; Circle: circular-pore titanium mesh; PEEK: polyetheretherketone plate.

### 3.2. Concentrated External Loading Condition

**Figure 5:**
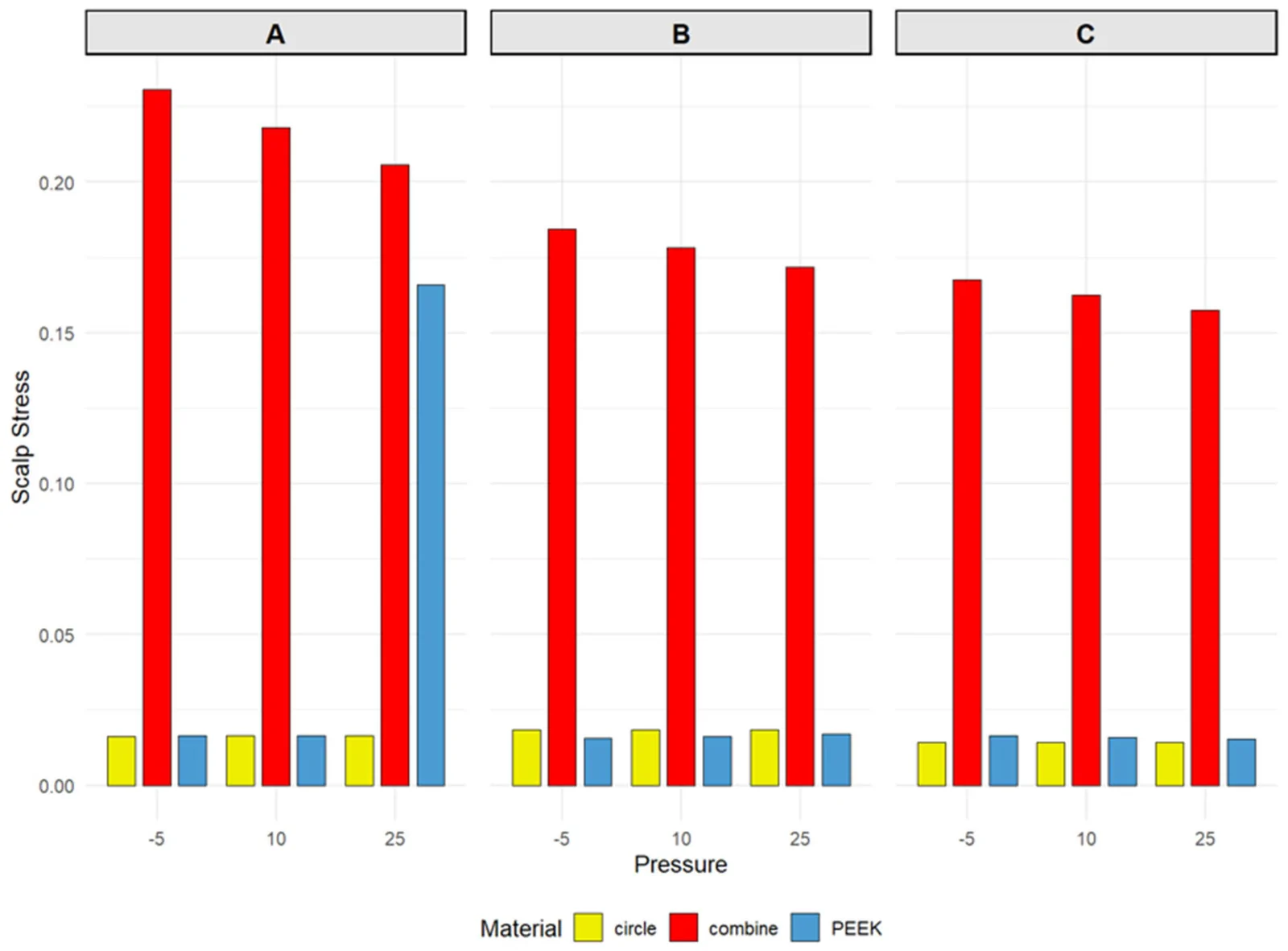
Scalp stress variations for the three cranioplasty materials under Mode 2 (concentrated external loading) at intracranial pressures of –5, 10, and 25 mmHg, with the external force applied at three designated positions: (**A**) (center of the implant), (**B**) (anterior to the center), and (**C**) (posterior to the center). Refer to Figure 2 for the detailed location of each position. Combine: Combine-pattern titanium mesh; Circle: circular-pore titanium mesh; PEEK: polyetheretherketone plate.

The locations of maximum scalp stress differed among the three materials. For both the Combine and PEEK materials, the maximum scalp stress occurred in the central region A, whereas for the Circle material, the peak stress was located in region B, anterior to the center. In the Combine group, the stress in region A reached 0.2307 MPa at –5 mmHg and decreased only slightly to 0.2056 MPa as ICP increased; the stresses in regions B and C ranged from 0.1845 MPa to 0.1718 MPa and from 0.1676 MPa to 0.1576 MPa, respectively, displaying a distinct spatial gradient. For the PEEK material under normal ICP (–5 and 10 mmHg), the stress in region A (0.01655 MPa and 0.01654 MPa) was slightly higher than that in region B (0.01554 MPa and 0.01616 MPa) and region C (0.01643 MPa and 0.01591 MPa), with minimal overall fluctuation. The stress in the Circle group remained within a narrow range of 0.014–0.019 MPa; however, its maximum value consistently appeared in region B (0.01852 MPa), while the values in regions A and C were approximately 0.0162–0.0164 MPa and 0.0141–0.0142 MPa, respectively (Figure 5).

Notably, the PEEK material exhibited an isolated stress peak of 0.166 MPa in region A at an ICP of 25 mmHg, a value approximately ten times higher than the stresses observed at other pressure levels for the same material (Figure 5). No similar abrupt change was observed in the Circle or Combine groups under the same condition.

**Figure 6:**
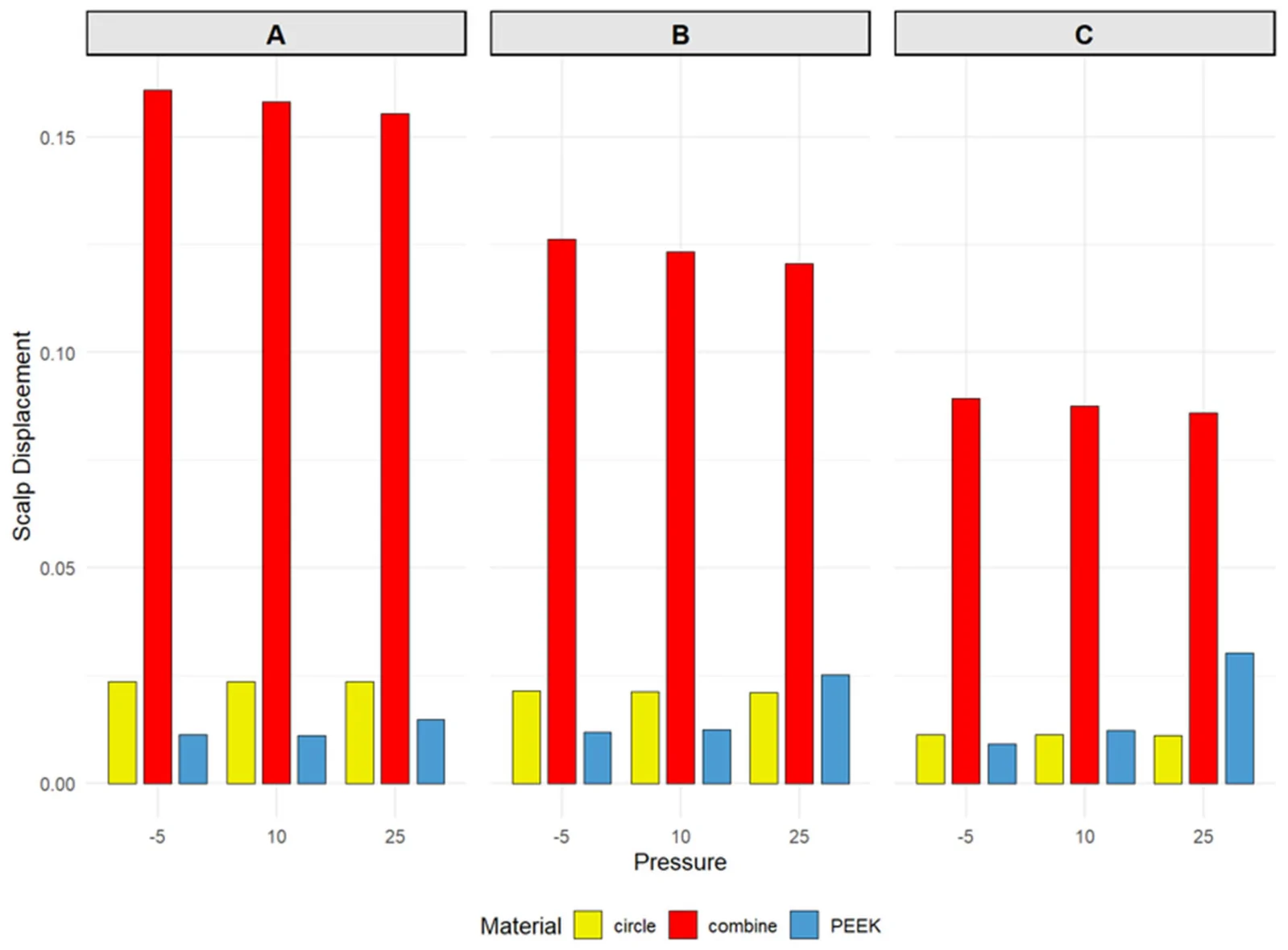
Scalp displacement variations for the three cranioplasty materials under Mode 2 (concentrated external loading) at intracranial pressures of –5, 10, and 25 mmHg, with the external force applied at three designated positions: (**A**) (center of the implant), (**B)** (anterior to the center), and (**C**) (posterior to the center). Refer to Figure 2 for the detailed location of each position. Combine: Combine-pattern titanium mesh; Circle: circular-pore titanium mesh; PEEK: polyetheretherketone plate.

The scalp displacement distribution was not entirely parallel to the stress distribution. The Combine titanium mesh still produced the largest displacement among the three materials, with the peak displacement consistently located in region A (Figure 6). Detailed data are provided in the Supplementary Materials (Tables S1–S3).

## 4. Discussion

This study employed finite element simulation to systematically compare the biomechanical responses of three cranial implant materials—Combine, Circle, and PEEK—under both uniform and localized loading conditions. The results demonstrate significant heterogeneity among the three materials in key evaluation metrics, including scalp stress, scalp displacement, implant stress, and implant displacement. These observed differences may have implications for understanding postoperative complications and may inform hypotheses for material selection.

Multiple prior investigations have consistently identified low ICP as a significant risk factor for titanium mesh exposure following cranioplasty. Kan et al. [26] noted that progressive scalp thinning is a common and frequently observed phenomenon after titanium mesh implantation, yet the precise underlying mechanism remains to be fully elucidated. Zheng et al. [12] further delineated the clinical association between low ICP and titanium mesh exposure: a significant alteration in ICP and a centripetal force on the porous titanium mesh are induced by the siphon effect generated by the ventriculoperitoneal shunt. Nonetheless, the biomechanical foundation underpinning these observations has hitherto lacked comprehensive characterization. The FEA presented in this study is the first to provide a comprehensive mechanical explanation of this mechanism. Under negative ICP conditions (e.g., −15 mmHg, −10 mmHg), scalp stress experienced by all three implant types was substantially elevated. At −15 mmHg, the Combine exhibited scalp stress values (0.0399 MPa) that were 20-fold higher than those of the Circle and 6-fold higher than those of PEEK. As ICP transitioned from negative to positive values, scalp stress decreased for all materials (reaching 0.0026 MPa for Combine at 30 mmHg). This trend strongly suggests that a low ICP environment significantly exacerbates the local mechanical compression exerted by the implant, particularly the Combine, on the overlying scalp, an effect that markedly diminishes upon restoration of normal or positive ICP levels. First, persistent traction exerted on the deep scalp tissues by the suction force may cause the relatively sharp edges of the titanium mesh to exert a chronic cutting action, which could facilitate the gradual migration of soft tissue through the mesh interstices toward the intracranial cavity. Consequently, the effective embedding depth of the mesh diminishes progressively and might manifest clinically as a peau d’orange appearance of the overlying skin or visible metallic contouring. Second, the suction force may act upon the microvascular network within the scalp flap, whereby tensile stress may induce luminal narrowing or outright occlusion of the microvessels, ultimately culminating in ischemic atrophy of the flap. The synergistic interplay between the aforementioned mechanical shearing and ischemic insult may contribute to the clinical outcome of titanium mesh exposure.

**Figure 7:**
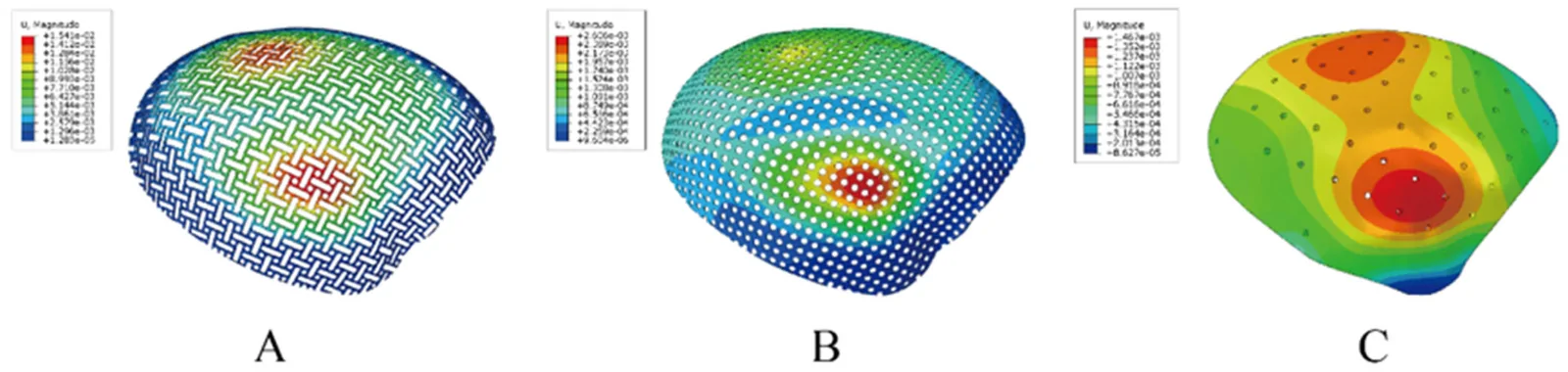
Displacement distribution for the three cranioplasty materials under Mode 1 (uniform external loading). The color scale ranges from blue (low displacement) to red (high displacement). All three materials exhibit a zone of relatively low displacement in the central area, and this low-displacement zone extends anteriorly and posteriorly from the center, forming a watershed-like pattern. According to Hooke’s law, displacement is positively correlated with the internal stress of the material. The watershed region exhibited low internal stress, whereas the zones directly superior and inferior to it showed the highest internal stress. (**A**): Combine-pattern titanium mesh; (**B**): circular-pore titanium mesh; (**C**): polyetheretherketone plate.

Clinically, titanium mesh exposure manifests in two principal patterns. The first involves exposure near the incision margins, typically associated with impaired wound healing or localized infection. The second pattern—central exposure in the setting of well-healed incisions—is often accompanied by widespread progressive scalp thinning and soft tissue atrophy [7,27]. However, our understanding of the mechanisms underlying these distinct patterns remains rudimentary, and dedicated research is sparse. Our findings indicate that under low ICP, negative pressure cannot effectively offset the uniform 50 N external load; instead, the two act additively, displacing the scalp and implant inward, increasing implant–scalp contact force and sharply amplifying local pressure. As described above, this sustained elevated stress may contribute to progressive scalp thinning and mesh exposure. This finding is broadly consistent with clinical observations by Wang et al. [28] and Mai et al. [29], who identified scalp thinning and soft tissue atrophy as significant risk factors for implant exposure and emphasized the close relationship between mesh exposure and the condition of the overlying scalp soft tissues. Our results provide a potential biomechanical explanation for the phenomenon of “diffuse scalp thinning–mesh exposure.” Prior finite element investigations of cranial implants have predominantly employed localized loading models, applying force via a cylindrical indenter directly onto the center or a specific site of the mesh to simulate focal impact or pressure [18,30,31,32]. However, such loading paradigms inadequately reflect the actual mechanical environment encountered by cranioplasty patients in a physiological context, wherein the reconstructed region is simultaneously subjected to constant external forces (e.g., head weight, postural variations, external contact) and dynamic internal pressure (ICP fluctuations). The approach used in this study more faithfully replicates clinical physiological conditions. This methodology also provides a quantitative characterization of the influence of ICP variation on the mechanical loading of the scalp and implant, offering new insights into the long-term mechanical adaptation following cranioplasty. In particular, the identification of markedly elevated scalp stress under low ICP and the phenomenon of central stress concentration may help identify biomechanical factors that warrant further investigation in clinical studies. Additionally, this loading paradigm revealed a distinct “watershed” effect regarding implant displacement and stress distribution. Specifically, a region of relatively lower stress and displacement was observed along the anteroposterior central axis of the implant, while the maximal implant stress and displacement occurred on either side of this watershed zone. Figure 7 shows the displacement patterns of the watershed effect for the three materials. According to Hooke’s Law, the greater the displacement, the greater the stress on the implant. Red represents the areas with significant displacement. Notably, the location of maximum scalp stress was diametrically opposed to that of the implant for the Combine and PEEK materials, with peak scalp stress consistently situated within the watershed region itself. Classical contact mechanics theory, consistent with the well-known Kirsch’s solution for stress concentration around a circular hole, indicates that sites of surface geometric discontinuity, such as edges and sharp corners, generate local stress concentrations in rigid–flexible body contact. In the present study, the region of the circular-hole titanium plate adjacent to the cranial base constitutes precisely this type of geometric discontinuity and constrained opening. When the soft tissue is compressed, it is extruded toward the hole, producing a contact stress concentration at the hole rim that far exceeds the macroscopic bending stress. This ultimately shifts the location of the peak stress from the central area to the region with surface irregularities. Clinically, exposure at the skull base is exceedingly rare, likely attributable to the robust vascular supply near the pedicle of the scalp flap and superior wound healing, despite marginally higher stress values compared to the central region in some instances.

To further probe the mechanical underpinnings of this central stress concentration, we applied localized loads at the central watershed region (Position A) and adjacent off-center sites (Positions B and C) (Figure 2), employing loading modalities consistent with prior studies. Our results demonstrate that under both uniform and localized loading conditions, the maximal scalp stress and displacement values were almost invariably concentrated at the central region (Position A) of the implant, with the notable exception of the PEEK implant, attributable to its inherent flexibility. This phenomenon can be explained by fundamental mechanical principles and boundary conditions. Firstly, the implant, secured to the skull margin via screws or fixation plates, can be approximated as an elastic thin plate with fixed peripheral support. When an external force is uniformly applied to the scalp overlying a rigid material, the reactive bending moments induced by the edge constraints result in maximal deflection and bending moment at the center of the plate. According to thin-plate theory, maximum stress and displacement always occur at the center. In the uniform loading scenario, the 50 N force applied vertically to the scalp is transmitted to the underlying implant. The central region, being farthest from all fixed edges, possesses the greatest degree of freedom for deformation, thereby exhibiting the highest amplitudes of stress and displacement. Secondly, under localized loading (50 N force applied via a 2 cm diameter cylinder), when force is applied at Position A, the load transfer path is longest, necessitating greater deformation to dissipate energy, resulting in maximal local stress and displacement. In contrast, when force is applied eccentrically at Position B or C, the load is closer to the ipsilateral fixed edge, which provides more direct mechanical support to constrain deformation. Consequently, peak stress and displacement values are generally lower than those observed during central loading. Furthermore, the scalp, being a soft tissue, exhibits a tendency to flow or redistribute away from the site of localized compression (akin to a hydraulic effect). Central loading allows for symmetric, omnidirectional tissue redistribution, whereas eccentric loading permits flow towards the nearest edge, where a portion of the pressure may be absorbed by the marginal structures, thereby mitigating the local peak. It should be noted that an exception was observed for the Circle at Position B, where scalp stress was marginally higher than at Position A. This minor deviation may be attributed to localized stress concentration effects around the circular pores under eccentric loading, though the overall difference was negligible. This central stress concentration may have potential clinical implications. For cranioplasty patients, particularly those receiving Combine, the central region may represent the zone of most severe mechanical loading on the scalp. These findings may suggest that future studies evaluate the importance of central scalp vascularity and thickness. Whether increasing central solid surface area would improve stress distribution needs further investigation.

In stark contrast to the Combine, the Circle exhibited the lowest and most stable scalp stress levels across all pressure conditions. Under uniform loading, the maximum stress in the Circle was located in the skull base region. Since the plate morphology in this area is undulating, the abnormal stress concentration may be attributable to the presence of large holes. After exclusion of the aberrant stress distribution at the skull base, the central scalp stress for the Circle model remained within a remarkably low range of 0.00063 MPa to 0.00199 MPa, approximating only 1/20th of the stress observed for the Combine model. Under localized loading conditions, its scalp stress remained stable between 0.014 MPa and 0.019 MPa, with minimal fluctuation. Moreover, the implant stress and displacement for the Circle model displayed a strong positive linear correlation (r = 0.998), consistent with linear elastic behavior and indicative of highly predictable mechanical responses. From a biomechanical perspective, this finding may be of interest, as in our study, the Circle implant showed lower scalp stress. Furthermore, by conducting a retrospective study, Slijepcevic et al. [33] identified free flap atrophy and inadequate soft tissue coverage as primary risk factors for implant exposure. The lower stress profile of Circle may reduce mechanical burden on the scalp and may be clinically favorable. Whether this translates into reduced clinical risk of scalp thinning or exposure requires clinical validation.

The PEEK implant exhibited mechanical characteristics alongside specific clinical advantages. Shash et al. [34] used FEA to compare PEEK and carbon-fiber-reinforced PEEK with traditional titanium implants, demonstrating large differences in deformation distribution patterns between PEEK and titanium implants. The elastic modulus of PEEK (approximately 3–4 GPa) closely approximates that of human cranial bone, which may help mitigate stress shielding and thereby reduce the risk of bone resorption. Radiologically, PEEK is radiolucent, producing no artifacts on CT or Magnetic Resonance Imaging (MRI) scans, offering a distinct advantage for patients requiring long-term neuroimaging surveillance. In this study, the PEEK material demonstrated excellent mechanical performance. Under uniformly distributed external loading and normal ICP, the PEEK implant exhibited minimal stress and displacement, and the overlying scalp experienced very low displacement and modest stress levels, comparable to those of the Circle, which showed the lowest scalp stress among the evaluated groups. Our results indicate that, relative to titanium plates, PEEK offers comparable or even superior mechanical behavior in terms of both implant response and scalp impact, while also reducing the stress shielding effect arising from the excessively high elastic modulus of titanium. PEEK therefore represents a promising candidate for next-generation cranioplasty materials. Of particular note, at Position A with an ICP of 25 mmHg, scalp stress for the PEEK model surged anomalously to 0.166 MPa, representing a significant departure from the expected response range. The micro-perforations in PEEK serve as local channels for pressure transmission. Under an ICP of 25 mmHg, the central opening—located far from the rigid support of the bone edge—allows the internal pressure to act directly on the inner surface of the scalp through the small hole, preloading the scalp with a strong outward hydraulic lift. When an external force is applied precisely over this same opening, the scalp at that site is simultaneously subjected to inward external mechanical pressure and outward internal hydraulic pressure. The superimposition of these two opposing loads generates a contact stress concentration at the opening edge that is substantially higher than that produced by either load alone [35,36,37]. In contrast, the lateral openings lie closer to the cranial support region, where the efficiency of internal pressure transmission is attenuated by the increased stiffness of the surrounding tissues; moreover, the peaks of the external force and internal pressure may be spatially misaligned. Consequently, a stress superimposition of comparable magnitude does not occur at these sites [35,36,37]. However, this phenomenon of increased scalp stress over the PEEK implant was observed only under intracranial hypertension in our results, as shown in Figure 4. Notably, sustained intracranial hypertension during the stable postoperative period of cranioplasty is uncommon.

This study is subject to several limitations. First, the simulation model relies on idealized geometry and material constitutive relationships, and does not fully capture individual anatomical variations, nonlinear tissue mechanics, or dynamic loading effects. Second, the constant 50 N external force, while based on commonly cited clinical parameters, simplifies the actual forces experienced by the head, which vary with posture and activity. Third, the precise cause of the anomalously high stress value observed for PEEK at Position A and 25 mmHg remains undetermined and warrants further investigation through experimental validation or more refined boundary condition analysis. Fourth, this investigation focused solely on static mechanical responses and did not assess long-term performance under fatigue loading, whereas clinical exposure is often a cumulative, long-term process. Kwiecien et al. [27] highlighted the gradual, progressive nature of scalp atrophy, suggesting that fatigue loading factors may be particularly relevant. Fifth, a sensitivity analysis evaluating the influence of fixation parameters (e.g., screw number, distribution, and tightening torque) was not performed; such factors could significantly impact stress distribution [38], particularly for flexible materials like PEEK. Future research should incorporate dynamic loading, fatigue assessment, patient-specific modeling, parametric fixation studies, and in vitro testing, and may explore 4D-printed elastomers [39] as compliant interfaces in hybrid cranioplasty constructs.

## 5. Conclusions

This study provides several biomechanical perspectives on cranioplasty. ICP significantly influenced scalp stress in the present model and may act as a mechanistic contributor to implant exposure, particularly central exposure despite well-healed incisions. The pronounced elevation in scalp stress observed with Combine may represent a key biomechanical mechanism underlying the “diffuse scalp thinning–mesh exposure” cascade, especially under low ICP conditions. The central region of the implant, being farthest from the fixed margins, was identified as the locus of peak stress and may warrant further investigation. Under the simulated conditions, Circle and PEEK exhibited more favorable stress distributions, suggesting potential biomechanical advantages. If Combine is used, increasing the solid surface area centrally may reduce local pressure in the model, but this needs further study. The Circle consistently demonstrated the lowest and most stable scalp stress across all simulated conditions, indicating a potentially favorable mechanical profile. PEEK offers advantages including radiolucency and favorable modulus matching, yet its nonlinear mechanical behavior and potential for anomalous high-stress responses under specific boundary conditions must be carefully considered; a comprehensive assessment of its risks and costs is essential for clinical application. Furthermore, the uniformly distributed loading paradigm introduced in this study offers a methodological framework for the biomechanical evaluation of cranial implant materials.

## Figures and Tables

**Table 1:** Correlation analysis between mechanical responses and intracranial pressure.

Material	Measure	Phase	r	*p*	Statistical Method
Combine	Implant Stress	Overall	−0.9981	<0.0001	Pearson
Combine	Implant Displacement	Overall	−0.9989	<0.0001	Pearson
Combine	Scalp Stress	Overall	−0.9996	<0.0001	Pearson
Combine	Scalp Displacement	Overall	−0.9989	<0.0001	Pearson
Circle	Implant Stress	Overall	−0.9905	<0.0001	Pearson
Circle	Implant Displacement	Overall	−0.9954	<0.0001	Pearson
Circle	Scalp Stress	−15 mmHg-Turn	−0.9923	<0.0001	Pearson
Circle	Scalp Stress	Turn to 30 mmHg	0.9891	<0.0001	Pearson
Circle	Scalp Displacement	Overall	−0.9954	<0.0001	Pearson
Peek	Implant Stress	Overall	−0.9862	<0.0001	Pearson
Peek	Implant Displacement	Overall	−0.9382	<0.0001	Pearson
Peek	Scalp Stress	−15 mmHg to Turn	−0.9824	<0.0001	Pearson
Peek	Scalp Stress	Turn to 30 mmHg	0.9823	<0.0001	Pearson
Peek	Scalp Displacement	Overall	−0.9784	<0.0001	Pearson

Correlation analysis of maximum scalp stress, maximum scalp displacement, maximum implant stress, and maximum implant displacement with intracranial pressure for the three materials. r: correlation coefficient; turn: turning point of the stress variation trend; overall: −15–30 mmHg. Combine: Combine-pattern titanium mesh; Circle: circular-pore titanium mesh; PEEK: polyetheretherketone plate.

**Table 2:** Multiplicative Comparison of Material Responses (Excluding Skull Base Region, Uniform Loading −15 to 15 mmHg).

Comparison	Metric	Multiplicative Factor Range
Combine/Circle	Scalp Stress	20–22
Combine/Circle	Implant Stress	6–7
Combine/PEEK	Scalp Stress	3–10
Combine/PEEK	Implant Stress	37–98
PEEK/Circle	Scalp Stress	2–6
PEEK/Circle	Implant Stress	0.07–0.16

## Data Availability

The data presented in this study are available on request from the corresponding author due to ethical restrictions imposed by the Ethics Committee of Huashan Hospital, Fudan University (Approval No. KY2024-1323), as the data contain de-identified patient CT images. The datasets generated and analyzed during the current study are available from the corresponding author upon reasonable request, subject to the same ethical restrictions. Data supporting Figure 4, Figure 5 and Figure 6 and Table 1 and Table 2 are provided in the Supplementary Materials. Figure 1, Figure 2 and Figure 7 are included in the related files submitted alongside the manuscript.

## Supplementary Materials

The following supporting information can be downloaded at: https://www.mdpi.com/article/10.3390/biomedicines14102348/s1, Table S1. Mode 2 stress/displacement, combined mesh, positions A–C. Table S2. Mode 2 stress/displacement, circular-pore mesh, positions A–C. Table S3. Mode 2 stress/displacement, PEEK, positions A–C. Table S4. Mode 1 stress/displacement, combined mesh. Table S5. Mode 1 stress/displacement, combined mesh, skull base excluded. Table S6. Mode 1 stress/displacement, circular-pore mesh. Table S7. Mode 1 stress/displacement, circular-pore mesh, skull base excluded. Table S8. Mode 1 stress/displacement, PEEK and fixation plate. Table S9. Mode 1 stress/displacement, PEEK, skull base excluded.

## Abbreviations

The following abbreviations are used in this manuscript:

3D	Three-dimensional
ANSA	Automatic Net Generation for Structural Analysis
C3D4	4-node linear tetrahedron
Circle	Circular-pore titanium mesh
Combine	Combine-pattern titanium mesh
CT	Computed Tomography
DICOM	Digital Imaging and Communications in Medicine
FEA	Finite Element Analysis
ICP	Intracranial Pressure
MIMICS	Materialise Interactive Medical Image Control System
MPC	Multi-Point Constraint
MRI	Magnetic Resonance Imaging
PACS	Picture Archiving and Communication System
PEEK	Polyetheretherketone plate
r	Correlation coefficient
S3	3-node linear triangular shell
STL	Standard Tessellation Language/Stereolithography

## References

[1] Zhang P., Fu X., Huang Y., Wound Repair Professional Committee of Chinese Medical Doctor Association (2024). Consensus on the prevention and repair of titanium mesh exposed wound after cranioplasty (2024 edition). Burns Trauma.

[2] Jonkergouw J., van de Vijfeijken S.E., Nout E., Theys T., Van de Casteele E., Folkersma H., Depauw P.R., Becking A.G. (2016). Outcome in patient-specific PEEK cranioplasty: A two-center cohort study of 40 implants. J. Craniomaxillofac. Surg..

[3] Punchak M., Chung L.K., Lagman C., Bui T.T., Lazareff J., Rezzadeh K., Jarrahy R., Yang I. (2017). Outcomes following polyetheretherketone (PEEK) cranioplasty: Systematic review and meta-analysis. J. Clin. Neurosci..

[4] Shah A.M., Jung H., Skirboll S. (2014). Materials used in cranioplasty: A history and analysis. Neurosurg. Focus.

[5] Kwiecien G.J., Rueda S., Couto R.A., Hashem A., Nagel S., Schwarz G.S., Zins J.E., Gastman B.R. (2018). Long-term outcomes of cranioplasty: Titanium mesh is not a long-term solution in high-risk patients. Ann. Plast. Surg..

[6] Mukherjee S., Thakur B., Haq I., Hettige S., Martin A.J. (2014). Complications of titanium cranioplasty—A retrospective analysis of 174 patients. Acta Neurochir..

[7] Maqbool T., Binhammer A., Binhammer P., Antonyshyn O.M. (2018). Risk factors for titanium mesh implant exposure following cranioplasty. J. Craniofac. Surg..

[8] Yeap M.C., Tu P.H., Liu Z.H., Hsieh P.C., Liu Y.T., Lee C.Y., Lai H.Y., Chen C.T., Huang Y.C., Wei K.C. (2019). Long-term complications of cranioplasty using stored autologous bone graft, three-dimensional polymethyl methacrylate, or titanium mesh after decompressive craniectomy: A single-center experience after 596 procedures. World Neurosurg..

[9] Thien A., King N.K., Ang B.T., Wang E., Ng I. (2015). Comparison of polyetheretherketone and titanium cranioplasty after decompressive craniectomy. World Neurosurg..

[10] Zanotti B., Zingaretti N., Verlicchi A., Robiony M., Alfieri A., Parodi P.C. (2016). Cranioplasty: Review of materials. J. Craniofac. Surg..

[11] Xie Y., Li S., Zhang T., Wang C., Cai X. (2020). Titanium mesh for bone augmentation in oral implantology: Current application and progress. Int. J. Oral Sci..

[12] Zheng R.Z., Qi Z.X., Lang L.J., Huang W.T., Chen J.Y., Zhu T.M., Wu X.H. (2025). Intracranial hypotension mechanism and implant retention procedure for patients with titanium mesh exposure. Oper. Neurosurg..

[13] Honeybul S., Ho K.M. (2011). Long-term complications of decompressive craniectomy for head injury. J. Neurotrauma.

[14] Krishnan P., De R. (2022). Sinking scalp flap with “implant patterning” after cranioplasty—An unusual complication of repair of a bony defect using a titanium mesh. Neurol. India.

[15] Huang M.T., Juan P.K., Chen S.Y., Wu C.J., Wen S.C., Cho Y.C., Huang M.S., Chou H.H., Ou K.L. (2019). The potential of the three-dimensional printed titanium mesh implant for cranioplasty surgery applications: Biomechanical behaviors and surface properties. Mater. Sci. Eng. C Mater. Biol. Appl..

[16] Kim T.Y., Lee M.H. (2026). Surface modification of cranioplasty implants to reduce postoperative cerebrospinal fluid collection: A preliminary clinical study. Korean J. Neurotrauma.

[17] Becker D.P., Miller J.D., Ward J.D., Greenberg R.P., Young H.F., Sakalas R. (1977). The outcome from severe head injury with early diagnosis and intensive management. J. Neurosurg..

[18] Ridwan-Pramana A., Marcián P., Borák L., Narra N., Forouzanfar T., Wolff J. (2017). Finite element analysis of 6 large PMMA skull reconstructions: A multi-criteria evaluation approach. PLoS ONE.

[19] Motherway J.A., Verschueren P., Van der Perre G., Vander Sloten J., Gilchrist M.D., Lim C.T., Goh J.C.H. (2010). The mechanical properties of cranial bone. Proceedings of the 6th World Congress of Biomechanics (WCB 2010) IFMBE Proceedings, Singapore, 1–6 August 2010.

[20] Mao H., Zhang L., Jiang B., Genthikatti V.V., Jin X., Zhu F., Makwana R., Gill A., Jandir G., Singh A. (2013). Development of a finite element human head model partially validated with thirty-five experimental cases. J. Biomech. Eng..

[21] Kleiven S. (2007). Predictors for traumatic brain injuries evaluated through accident reconstructions. Stapp Car Crash J..

[22] Nguyen Huu L., Hoang Hong H., Nguyen T.H., Hai D.N., Ngo Trung N., Kim C.N.T. (2025). Numerical modeling and evaluation of the biomechanical performance and stability of cranial implants with varying thicknesses and biomaterials. Eng. Res. Express.

[23] Lu Y., Bai J., Liu Y., Shu Y. (2025). Head injury risk prediction for vulnerable road users based on Chinese adult male head data. Sci. Rep..

[24] Mian S.H., Moiduddin K., Elseufy S.M., Alkhalefah H. (2022). Adaptive mechanism for designing a personalized cranial implant and its 3D printing using PEEK. Polymers.

[25] Persson J., Helgason B., Engqvist H., Ferguson S.J., Persson C. (2018). Stiffness and strength of cranioplastic implant systems in comparison to cranial bone. J. Craniomaxillofac. Surg..

[26] Kan D., He X., Liu B., Yang C., Zou Y. (2023). Full-thickness skin regeneration beneath the exposed titanium mesh in cranioplasty: Two cases report. Medicine.

[27] Kwiecien G.J., Sinclair N., Coombs D.M., Djohan R.S., Mihal D., Zins J.E. (2020). Long-term effect of cranioplasty on overlying scalp atrophy. Plast. Reconstr. Surg. Glob. Open.

[28] Wang W., Vincent A., Bahrami A., Shokri T., Inman J., Ducic Y. (2020). Progressive scalp thinning over mesh cranioplasty and the role of lipotransfer. Laryngoscope.

[29] Bui M.A., Pham N.H., Vu T.T. (2025). Saving exposed titanium mesh cranioplasty using adipocutaneous anterolateral thigh flap: A case series. JPRAS Open.

[30] Ulmeanu M.E., Doicin C.V., Mates I., Murzac R., Davitoiu D. (2020). Impact FEA simulation and analysis of custom-made cranial implants. Rev. Chim..

[31] Huys S.E.F., Van Gysel A., Mommaerts M.Y., Vander Sloten J. (2021). Evaluation of patient-specific cranial implant design using finite element analysis. World Neurosurg..

[32] Siegel A., Sauter-Starace F., Laporte S. (2022). Insights into the mechanical interaction between an active cranial implant and the skull subjected to moderate impact loadings. J. Mech. Behav. Biomed. Mater..

[33] Slijepcevic A.A., Scott B., Lilly G.L., Young G., Taghizadeh F., Li R., Petrisor D., Pipkorn P., Rich J., Jackson R. (2023). Outcomes of cranioplasty reconstructions: Review of cranioplasty implants and free flap coverage variables that affect implant exposure. Laryngoscope.

[34] Shash Y.H. (2025). Correction: Cranial reconstruction utilizing polymeric implants in two different designs: Finite element investigation. BMC Musculoskelet. Disord..

[35] Bogu V.P., Kumar Y.R., Kumar Khanara A. (2017). Modelling and structural analysis of skull/cranial implant: Beyond mid-line deformities. Acta Bioeng. Biomech..

[36] Cuc N.T.K., Hung P.D., Duc B.M., Anh N.H., Khang N.V., Hoang N.Q., Ceccarelli M. (2022). Mechanical evaluation of the large cranial implant using finite elements method. Advances in Asian Mechanism and Machine Science.

[37] Mejía Rodríguez M., González-Estrada O.A., Villegas-Bermúdez D.F. (2024). Finite element analysis of patient-specific cranial implants under different design parameters for material selection. Designs.

[38] Ridwan-Pramana A., Marcián P., Borák L., Narra N., Forouzanfar T., Wolff J. (2016). Structural and mechanical implications of PMMA implant shape and interface geometry in cranioplasty—A finite element study. J. Craniomaxillofac. Surg..

[39] Bayati A., Rahmatabadi D., Khajepour M., Baniassadi M., Abrinia K., Bodaghi M., Baghani M. (2025). 4D printing of composite thermoplastic elastomers for super-stretchable soft artificial muscles. J. Appl. Polym. Sci..

