# Supplementary material for: The Biomechanical Mechanism of Cranial Implant Exposure Following Decreased Intracranial Pressure: A Finite Element Analysis Study
Source: Biomedicines. 2026 Oct 15;14(10):2348. doi: 10.3390/biomedicines14102348 (PMC13702183; doi:10.3390/biomedicines14102348)
Supplement: Supplementary file 1 [file biomedicines-14-02348-s001.zip › biomedicines-4600304-supplementary.pdf]

Table S1. Mode 2 stress/displacement, combine mesh, positions A–C.

combine-pattern titanium mesh						
position: A						
ICP(mmHg)	Implant Stress (MPa)	Implant Displacement (mm)	Cranial stress (MPa)	Cranial displacement (mm)	Scalp Stress (MPa)	Scalp Displacement (mm)
-5	93.42	0.1608	1.405	0.008012	0.2307	0.1609
10	90.43	0.1579	1.397	0.007596	0.2181	0.1581
25	88.71	0.155	1.39	0.007185	0.2056	0.1554
position: B						
ICP(mmHg)	Implant Stress (MPa)	Implant Displacement (mm)	Cranial stress (MPa)	Cranial displacement (mm)	Scalp Stress (MPa)	Scalp Displacement (mm)
-5	140.3	0.1263	3.264	0.005222	0.1845	0.1263
10	137.4	0.1234	3.136	0.00495	0.1782	0.1234
25	134.5	0.1205	3.008	0.00468	0.1718	0.1205
position: C						
ICP(mmHg)	Implant Stress (MPa)	Implant Displacement (mm)	Cranial stress (MPa)	Cranial displacement (mm)	Scalp Stress (MPa)	Scalp Displacement (mm)
-5	95.08	0.08753	2.196	0.005221	0.1676	0.08924
10	94.24	0.08594	2.011	0.004832	0.1626	0.08759
25	93.42	0.08434	1.831	0.00446	0.1576	0.08595

Implant Stress, Implant Displacement, Cranial stress, Cranial displacement, Scalp Stress and Scalp Displacement variations for the combine-pattern titanium mesh under Mode 2 (concentrated external loading) at intracranial pressures of –5, 10, and 25 mmHg, with the external force applied at three designated positions: A (center of the implant), B (anterior to the center), and C (posterior to the center).

Table S2. Mode 2 stress/displacement, circular-pore mesh, positions A–C.

circular-pore titanium mesh						
position: A						
ICP(mmHg)	Implant Stress (MPa)	Implant Displacement (mm)	Cranial stress (MPa)	Cranial displacement (mm)	Scalp Stress (MPa)	Scalp Displacement (mm)
-5	25.65	0.02369	0.4304	0.001631	0.01619	0.02366
10	25.72	0.02366	0.4264	0.0016	0.0163	0.02363
25	25.79	0.02362	0.424	0.0157	0.01641	0.02362
position: B						
ICP(mmHg)	Implant Stress (MPa)	Implant Displacement (mm)	Cranial stress (MPa)	Cranial displacement (mm)	Scalp Stress (MPa)	Scalp Displacement (mm)
-5	24.52	0.01973	0.9543	0.001444	0.01852	0.02139
10	24.32	0.01959	0.9421	0.001437	0.01852	0.02123
25	24.11	0.01946	0.9304	0.001431	0.01852	0.02107
position: C						
ICP(mmHg)	Implant Stress (MPa)	Implant Displacement (mm)	Cranial stress (MPa)	Cranial displacement (mm)	Scalp Stress (MPa)	Scalp Displacement (mm)
-5	20.27	0.01091	0.5471	0.001187	0.0141	0.01127
10	20.15	0.01087	0.5249	0.001159	0.01412	0.01121
25	20.03	0.01083	0.5038	0.001147	0.01415	0.01115

Implant Stress, Implant Displacement, Cranial stress, Cranial displacement, Scalp Stress and Scalp Displacement variations for the circular-pore titanium mesh under Mode 2 (concentrated external loading) at intracranial pressures of –5, 10, and 25 mmHg, with the external force applied at three designated positions: A (center of the implant), B (anterior to the center), and C (posterior to the center).

Table S3. Mode 2 stress/displacement, PEEK, positions A–C.

polyetheretherketone plate						
position: A						
ICP(mmHg)	Implant Stress (MPa)	Implant Displacement (mm)	Cranial stress (MPa)	Cranial displacement (mm)	Scalp Stress (MPa)	Scalp Displacement (mm)
-5	1.016	0.007806	1.108	0.001678	0.01655	0.01127
10	0.999	0.00768	1.272	0.001744	0.01654	0.01105
25	0.9859	0.01475	1.572	0.001868	0.166	0.01475
position: B						
ICP(mmHg)	Implant Stress (MPa)	Implant Displacement (mm)	Cranial stress (MPa)	Cranial displacement (mm)	Scalp Stress (MPa)	Scalp Displacement (mm)
-5	1.012	0.007849	3.365	0.001878	0.01554	0.01181
10	1.162	0.01236	3.851	0.00206	0.01616	0.01236
25	1.296	0.02514	4.34	0.002273	0.01685	0.02514
position: C						
ICP(mmHg)	Implant Stress (MPa)	Implant Displacement (mm)	Cranial stress (MPa)	Cranial displacement (mm)	Scalp Stress (MPa)	Scalp Displacement (mm)
-5	1.085	0.005742	2.844	0.001846	0.01643	0.009108
10	1.63	0.01218	3.198	0.002139	0.01591	0.01218
25	2.247	0.03027	3.384	0.003219	0.01541	0.03027

Implant Stress, Implant Displacement, Cranial stress, Cranial displacement, Scalp Stress and Scalp Displacement variations for the polyetheretherketone plate under Mode 2 (concentrated external loading) at intracranial pressures of –5, 10, and 25 mmHg, with the external force applied at three designated positions: A (center of the implant), B (anterior to the center), and C (posterior to the center).

Table S4. Mode 1 stress/displacement, combine mesh.

combine-pattern titanium mesh						
ICP(mmHg)	Implant Stress (MPa)	Implant Displacement (mm)	Cranial stress (MPa)	Cranial displacement (mm)	Scalp Stress (MPa)	Scalp Displacement (mm)
-15	19.6565	0.026956	0.962987	0.003023	0.0399	0.026976
-14.265	19.33366	0.026512	0.94881	0.003003	0.03926	0.026531
-13.53	19.0108	0.026068	0.934635	0.002984	0.03862	0.026086
-12.795	18.68794	0.025623	0.920464	0.002964	0.03798	0.025641
-12.06	18.36507	0.025179	0.906298	0.002945	0.03734	0.025196
-11.325	18.04221	0.024735	0.892137	0.002925	0.0367	0.024752
-10.59	17.71935	0.024291	0.87798	0.002906	0.03606	0.024307
-9.855	17.3965	0.023847	0.863828	0.002886	0.03542	0.023862
-9.12	17.07364	0.023402	0.849681	0.002867	0.03478	0.023417
-8.385	16.75078	0.022958	0.83554	0.002847	0.03414	0.022972
-7.65	16.42792	0.022514	0.821405	0.002828	0.0335	0.022527
-6.915	16.10507	0.02207	0.807276	0.002808	0.03286	0.022083
-6.18	15.78222	0.021626	0.793153	0.002789	0.03222	0.021638
-5.445	15.45936	0.021182	0.779037	0.002769	0.03157	0.021193
-4.71	15.13651	0.020737	0.764929	0.00275	0.03093	0.020748
-3.975	14.81366	0.020293	0.750827	0.00273	0.03029	0.020303
-3.24	14.49081	0.019849	0.736734	0.002711	0.02965	0.019858
-2.505	14.16796	0.019405	0.722649	0.002692	0.02901	0.019414

-1.77	13.84512	0.018961	0.708573	0.002672	0.02837	0.018969
-1.035	13.52227	0.018517	0.694507	0.002653	0.02773	0.018524
-0.3	13.19943	0.018072	0.680451	0.002634	0.02709	0.018079
0.435	12.87659	0.017628	0.666405	0.002614	0.02645	0.017634
1.17	12.55375	0.017184	0.652371	0.002595	0.02581	0.017189
1.905	12.23091	0.01674	0.638349	0.002576	0.02517	0.016745
2.64	11.90808	0.016296	0.62434	0.002557	0.02453	0.0163
3.375	11.58525	0.015851	0.610345	0.002537	0.02389	0.015856
4.11	11.26242	0.015407	0.596365	0.002518	0.02325	0.015412
4.845	10.93959	0.014963	0.5824	0.002499	0.02261	0.014967
5.58	10.61677	0.014519	0.568453	0.00248	0.02197	0.014523
6.315	10.29395	0.014075	0.554524	0.002461	0.02133	0.014078
7.05	9.971141	0.013631	0.540615	0.002442	0.02069	0.013634
7.785	9.648332	0.013186	0.526727	0.002422	0.02005	0.01319
8.52	9.325527	0.012742	0.512862	0.002403	0.01941	0.012745
9.255	9.002728	0.012298	0.499022	0.002384	0.01877	0.012301
9.99	8.679936	0.011854	0.485208	0.002365	0.01813	0.011856
10.725	8.35715	0.01141	0.471425	0.002346	0.01749	0.011412
11.46	8.034372	0.010966	0.457673	0.002327	0.01685	0.010968
12.195	7.711602	0.010521	0.443956	0.002308	0.01621	0.010523
12.93	7.388842	0.010077	0.430277	0.002289	0.01557	0.010079
13.665	7.066094	0.009634	0.416641	0.00227	0.01493	0.009635

14.4	6.743358	0.009191	0.40305	0.002252	0.01429	0.009191
15.135	6.420637	0.008748	0.389511	0.002233	0.01365	0.008748
15.87	6.097932	0.008304	0.376027	0.002214	0.01301	0.008305
16.605	5.775248	0.007861	0.362607	0.002195	0.01237	0.007861
17.34	5.452588	0.007431	0.360764	0.002176	0.01174	0.007438
18.075	5.129955	0.007068	0.361554	0.002157	0.0111	0.007074
18.81	4.807355	0.006705	0.362345	0.002139	0.01046	0.006711
19.545	4.484797	0.006343	0.363138	0.00212	0.009823	0.006349
20.28	4.162289	0.005982	0.363931	0.002101	0.009187	0.005988
21.015	3.839844	0.005623	0.364725	0.002083	0.00855	0.005628
21.75	3.51748	0.005264	0.365519	0.002064	0.007915	0.00527
22.485	3.210979	0.004908	0.366315	0.002046	0.007281	0.004913
23.22	3.034737	0.004553	0.367111	0.002028	0.006648	0.004559
23.955	2.858708	0.004201	0.367908	0.002011	0.006016	0.004207
24.69	2.682933	0.003853	0.368706	0.001994	0.005387	0.003858
25.425	2.507468	0.003508	0.369505	0.001976	0.004877	0.003513
26.16	2.332385	0.00317	0.370304	0.001959	0.00441	0.003175
26.895	2.157778	0.002839	0.371105	0.001941	0.003947	0.002844
27.63	1.983778	0.002519	0.371906	0.001924	0.00349	0.002524
28.365	1.861016	0.002215	0.372708	0.001907	0.00309	0.00222
29.1	1.790686	0.001943	0.37351	0.00189	0.002801	0.001945
29.835	1.722178	0.00187	0.374313	0.001873	0.002627	0.00187

30	1.707062	0.001867	0.374494	0.001869	0.002595	0.001867

Implant Stress, Implant Displacement, Cranial stress, Cranial displacement, Scalp Stress and Scalp Displacement variations for the combine-pattern titanium mesh under Mode 1.

Table S5. Mode 1 stress/displacement, combine mesh, skull base excluded.

combine-pattern titanium mesh				
ICP(mmHg)	Implant Stress (MPa)	Implant Displacement (mm)	Scalp Stress (MPa)	Scalp Displacement (mm)
-15	18.23974	0.023633	0.039904	0.023642
-14.265	17.93876	0.023263	0.039263	0.023272
-13.53	17.63775	0.022894	0.038622	0.022902
-12.795	17.33675	0.022524	0.037981	0.022532
-12.06	17.03574	0.022155	0.037341	0.022162
-11.325	16.73475	0.021785	0.0367	0.021792
-10.59	16.43375	0.021416	0.036059	0.021423
-9.855	16.13275	0.021046	0.035419	0.021053
-9.12	15.83176	0.020677	0.034778	0.020683
-8.385	15.53077	0.020308	0.034137	0.020314
-7.65	15.22978	0.019938	0.033497	0.019944
-6.915	14.92879	0.019569	0.032856	0.019575
-6.18	14.62781	0.0192	0.032215	0.019205
-5.445	14.32683	0.018831	0.031575	0.018836
-4.71	14.02585	0.018461	0.030934	0.018466
-3.975	13.72488	0.018092	0.030294	0.018097
-3.24	13.4239	0.017723	0.029653	0.017728
-2.505	13.12294	0.017354	0.029013	0.017358
-1.77	12.82197	0.016985	0.028372	0.016989
-1.035	12.52102	0.016616	0.027732	0.01662
-0.3	12.22006	0.016247	0.027091	0.016252
0.435	11.91911	0.015878	0.026451	0.015883
1.17	11.61817	0.01551	0.02581	0.015514
1.905	11.31723	0.015141	0.02517	0.015145
2.64	11.0163	0.014772	0.02453	0.014777
3.375	10.71538	0.014404	0.023889	0.014408
4.11	10.41446	0.014035	0.023249	0.01404
4.845	10.11355	0.013667	0.022609	0.013671

5.58	9.812651	0.013298	0.021968	0.013303
6.315	9.51176	0.01293	0.021328	0.012935
7.05	9.210879	0.012562	0.020688	0.012567
7.785	8.910011	0.012194	0.020048	0.012199
8.52	8.609156	0.011826	0.019408	0.011831
9.255	8.308314	0.011459	0.018768	0.011463
9.99	8.007489	0.011091	0.018128	0.011096
10.725	7.706681	0.010724	0.017489	0.010729
11.46	7.405894	0.010357	0.016849	0.010362
12.195	7.105128	0.00999	0.016209	0.009995
12.93	6.804388	0.009623	0.01557	0.009629
13.665	6.503676	0.009257	0.01493	0.009263
14.4	6.202997	0.008891	0.014291	0.008897
15.135	5.902356	0.008525	0.013652	0.008531
15.87	5.601758	0.00816	0.013013	0.008166
16.605	5.301212	0.007795	0.012375	0.007802
17.34	5.000727	0.007431	0.011736	0.007438
18.075	4.700313	0.007068	0.011098	0.007074
18.81	4.399985	0.006705	0.010461	0.006711
19.545	4.099761	0.006343	0.009823	0.006349
20.28	3.799668	0.005982	0.009187	0.005988
21.015	3.56397	0.005623	0.00855	0.005628
21.75	3.387399	0.005264	0.007915	0.00527
22.485	3.210979	0.004908	0.007281	0.004913
23.22	3.034738	0.004553	0.006648	0.004559
23.955	2.858708	0.004201	0.006016	0.004207
24.69	2.682933	0.003853	0.005387	0.003858
25.425	2.507468	0.003508	0.004877	0.003513
26.16	2.332385	0.00317	0.00441	0.003175
26.895	2.157778	0.002839	0.003947	0.002844
27.63	1.983778	0.002519	0.00349	0.002524
28.365	1.828158	0.002215	0.00309	0.00222
29.1	1.731449	0.001943	0.002801	0.001945
29.835	1.694552	0.00187	0.002627	0.00187
30	1.686607	0.001867	0.002595	0.001867

Implant Stress, Implant Displacement, Scalp Stress and Scalp Displacement variations for the combine-pattern titanium mesh under Mode 1.Stress and displacement of the remaining cranioplasty implant and overlying scalp following resection of the portions adjacent to the anterior skull base. Given that the uneven surface of the implant adjacent to the skull base could lead to aberrant stress and displacement, this region was excluded for subsequent analysis.

Table S6. Mode 1 stress/displacement, circular-pore mesh.

circular-pore titanium mesh						
ICP(mmHg)	Implant Stress (MPa)	Implant Displacement (mm)	Cranial stress (MPa)	Cranial displacement (mm)	Scalp Stress (MPa)	Scalp Displacement (mm)
-15	3.62666	0.004257	0.32633	0.000992	0.00249	0.004277
-14.265	3.57068	0.004193	0.32597	0.00099	0.002415	0.004213
-13.53	3.514698	0.004129	0.325614	0.000988	0.00234	0.004149
-12.795	3.458718	0.004066	0.325263	0.000986	0.002265	0.004084
-12.06	3.40274	0.004002	0.324916	0.000984	0.002191	0.00402
-11.325	3.346764	0.003938	0.324575	0.000982	0.002116	0.003956
-10.59	3.290791	0.003875	0.324239	0.00098	0.002046	0.003892
-9.855	3.23482	0.003811	0.323908	0.000978	0.002006	0.003828
-9.12	3.178852	0.003748	0.323583	0.000977	0.001966	0.003764
-8.385	3.122887	0.003684	0.323262	0.000975	0.001926	0.0037
-7.65	3.066925	0.00362	0.322946	0.000973	0.001886	0.003635
-6.915	3.010966	0.003557	0.322635	0.000971	0.001846	0.003571
-6.18	2.95501	0.003493	0.32233	0.000969	0.001806	0.003507
-5.445	2.899057	0.00343	0.32203	0.000967	0.001765	0.003443
-4.71	2.843108	0.003366	0.321734	0.000965	0.001725	0.003379
-3.975	2.787163	0.003303	0.321444	0.000963	0.001865	0.003315
-3.24	2.731222	0.00324	0.32116	0.000961	0.001645	0.003251
-2.505	2.675286	0.003176	0.32088	0.00096	0.001605	0.003187
-1.77	2.61935	0.003113	0.32060	0.000958	0.00156	0.003122

	4		6		5	
-1.035	2.563427	0.003049	0.320336	0.000956	0.001525	0.003058
-0.3	2.507505	0.002986	0.320073	0.000954	0.001485	0.002994
0.435	2.451589	0.002923	0.319814	0.000952	0.001444	0.00293
1.17	2.395679	0.002859	0.31956	0.00095	0.001404	0.002866
1.905	2.339775	0.002796	0.319312	0.000949	0.001364	0.002802
2.64	2.283879	0.002732	0.319069	0.000947	0.001324	0.002738
3.375	2.227989	0.002669	0.318832	0.000945	0.001284	0.002674
4.11	2.172108	0.002606	0.3186	0.000943	0.001244	0.00261
4.845	2.116235	0.002542	0.318373	0.000941	0.001204	0.002545
5.58	2.060372	0.002479	0.318151	0.00094	0.001164	0.002482
6.315	2.004518	0.002415	0.317935	0.000938	0.001123	0.002419
7.05	1.948676	0.002352	0.317724	0.000936	0.001083	0.002356
7.785	1.892845	0.002289	0.317519	0.000934	0.001085	0.002293
8.52	1.837028	0.002225	0.317318	0.000932	0.001094	0.00223
9.255	1.781224	0.002162	0.317124	0.000931	0.001103	0.002166
9.99	1.725436	0.002099	0.316934	0.000929	0.001113	0.002103
10.725	1.669665	0.002035	0.31675	0.000927	0.001122	0.00204
11.46	1.613912	0.001972	0.316572	0.000925	0.001132	0.001977
12.195	1.558181	0.001909	0.316399	0.000924	0.001142	0.001914
12.93	1.502472	0.001846	0.316231	0.000922	0.001183	0.001851
13.665	1.446789	0.001783	0.316069	0.00092	0.001253	0.001788

14.4	1.391135	0.00172	0.315912	0.000918	0.001325	0.001725
15.135	1.335512	0.001657	0.31576	0.000917	0.001399	0.001661
15.87	1.279927	0.001594	0.315614	0.000915	0.001473	0.001598
16.605	1.224382	0.001531	0.315474	0.000913	0.001548	0.001535
17.34	1.168885	0.001468	0.315339	0.000911	0.001624	0.001472
18.075	1.113441	0.001406	0.315209	0.00091	0.0017	0.001409
18.81	1.058061	0.001343	0.315085	0.000908	0.001777	0.001346
19.545	1.002753	0.001281	0.314967	0.000906	0.001854	0.001283
20.28	0.947531	0.001219	0.314854	0.000905	0.001932	0.00122
21.015	0.906214	0.001158	0.314746	0.000904	0.00201	0.001157
21.75	0.898593	0.001096	0.314644	0.000906	0.002089	0.0011
22.485	0.891003	0.001062	0.314547	0.000907	0.002168	0.001074
23.22	0.883445	0.001038	0.314456	0.000908	0.002247	0.001047
23.955	0.875919	0.001014	0.314371	0.00091	0.002326	0.001022
24.69	0.868426	0.000993	0.31429	0.000912	0.002406	0.000997
25.425	0.860968	0.000973	0.314216	0.000914	0.002486	0.000973
26.16	0.853545	0.000956	0.314147	0.000916	0.002565	0.000957
26.895	0.846158	0.000941	0.314083	0.000918	0.002646	0.000942
27.63	0.840979	0.000929	0.314025	0.000921	0.002726	0.000929
28.365	0.837565	0.000919	0.313973	0.000923	0.002806	0.000918
29.1	0.834158	0.000909	0.313926	0.000926	0.002887	0.000908
29.835	0.830761	0.000901	0.313884	0.000929	0.002967	0.000901

30	0.829999	0.0009	0.313876	0.000929	0.002985	0.000901

Implant Stress, Implant Displacement, Cranial stress, Cranial displacement, Scalp Stress and Scalp Displacement variations for the circular-pore titanium mesh under Mode 1.

Table S7. Mode 1 stress/displacement, circular-pore mesh, skull base excluded.

circular-pore titanium mesh				
ICP(mmHg)	Implant Stress (MPa)	Implant Displacement (mm)	Scalp Stress (MPa)	Scalp Displacement (mm)
-15	2.557424	0.002703	0.001986	0.002746
-14.265	2.516785	0.002668	0.00195	0.002711
-13.53	2.47615	0.002633	0.001915	0.002676
-12.795	2.43552	0.002599	0.00188	0.00264
-12.06	2.394896	0.002564	0.001844	0.002605
-11.325	2.354279	0.00253	0.001809	0.00257
-10.59	2.313669	0.002495	0.001773	0.002535
-9.855	2.273066	0.002461	0.001738	0.0025
-9.12	2.232471	0.002426	0.001703	0.002465
-8.385	2.191884	0.002392	0.001667	0.002431
-7.65	2.151305	0.002357	0.001632	0.002396
-6.915	2.110735	0.002323	0.001596	0.002361
-6.18	2.070175	0.002289	0.001561	0.002326
-5.445	2.029625	0.002255	0.001526	0.002292
-4.71	1.989086	0.002221	0.00149	0.002257
-3.975	1.948559	0.002187	0.001455	0.002222
-3.24	1.908043	0.002153	0.001419	0.002188
-2.505	1.86754	0.002119	0.001384	0.002154
-1.77	1.827052	0.002085	0.001349	0.002119
-1.035	1.786578	0.002051	0.001313	0.002085
-0.3	1.746119	0.002018	0.001278	0.002051
0.435	1.705678	0.001984	0.001243	0.002017
1.17	1.665255	0.00195	0.001207	0.001983
1.905	1.624852	0.001917	0.001172	0.001949
2.64	1.584469	0.001884	0.001137	0.001915
3.375	1.544109	0.001851	0.001101	0.001881
4.11	1.503774	0.001817	0.001066	0.001848
4.845	1.463466	0.001784	0.00103	0.001814
5.58	1.423186	0.001752	0.000995	0.001781

6.315	1.382938	0.001719	0.00096	0.001748
7.05	1.342725	0.001686	0.000924	0.001715
7.785	1.302549	0.001654	0.000889	0.001682
8.52	1.262414	0.001621	0.00086	0.001649
9.255	1.222324	0.001589	0.000832	0.001616
9.99	1.182285	0.001557	0.000804	0.001584
10.725	1.142301	0.001526	0.000776	0.001552
11.46	1.102379	0.001495	0.000749	0.00152
12.195	1.062525	0.001464	0.000723	0.001488
12.93	1.022749	0.001433	0.000698	0.001456
13.665	0.999299	0.001403	0.000676	0.001425
14.4	0.979617	0.001373	0.00067	0.001393
15.135	0.968183	0.001343	0.000665	0.001363
15.87	0.960347	0.001313	0.000659	0.001332
16.605	0.952534	0.001284	0.000653	0.001302
17.34	0.944747	0.001254	0.000648	0.001272
18.075	0.936986	0.001226	0.000642	0.001242
18.81	0.929251	0.001197	0.000636	0.001213
19.545	0.921544	0.001169	0.000631	0.001184
20.28	0.913864	0.001142	0.000625	0.001156
21.015	0.906214	0.001115	0.00062	0.001128
21.75	0.898593	0.001088	0.00062	0.0011
22.485	0.891003	0.001062	0.000629	0.001074
23.22	0.883445	0.001038	0.000639	0.001047
23.955	0.875919	0.001014	0.000648	0.001022
24.69	0.868426	0.000993	0.000657	0.000997
25.425	0.860968	0.000973	0.000667	0.000973
26.16	0.853545	0.000956	0.000676	0.000957
26.895	0.846158	0.000941	0.000686	0.000942
27.63	0.840979	0.000929	0.000695	0.000929
28.365	0.837564	0.000919	0.000705	0.000918
29.1	0.834158	0.000909	0.000714	0.000908
29.835	0.830761	0.000901	0.000723	0.000901
30	0.829999	0.0009	0.000726	0.000901

Implant Stress, Implant Displacement, Scalp Stress and Scalp Displacement variations for the circular-pore titanium mesh under Mode 1. Stress and displacement of the remaining cranioplasty implant and overlying scalp following resection of the portions adjacent to the anterior skull base. Given that the uneven surface of the implant adjacent to the skull base could lead to aberrant stress and displacement, this region was excluded for subsequent analysis.

Table S8. Mode 1 stress/displacement, PEEK and fixation plate.

polyetheretherketone plate								
ICP(mm Hg)	Implant Stress (MPa)	Implant Displacement (mm)	Cranial stress (MPa)	Cranial displacement (mm)	Scalp Stress (MPa)	Scalp Displacement (mm)	Itanium fixation plate Stress (MPa)	Itanium fixation plate Displacement (mm)
-15	0.450883	0.002186	0.529517	0.00106	0.00651	0.00228	1.076592	0.001155
-14.265	0.445288	0.002158	0.528892	0.001058	0.006294	0.002252	1.06571	0.001151
-13.53	0.439664	0.00213	0.528294	0.001057	0.006079	0.002225	1.054795	0.001147
-12.795	0.434025	0.002102	0.527716	0.001055	0.005863	0.002197	1.043895	0.001143
-12.06	0.428378	0.002073	0.527156	0.001053	0.005648	0.002169	1.033047	0.001138
-11.325	0.422713	0.002045	0.526607	0.001052	0.005433	0.002142	1.030402	0.001134
-10.59	0.417038	0.002018	0.526075	0.001051	0.005218	0.002114	1.032611	0.00113
-9.855	0.411346	0.00199	0.525561	0.001049	0.005003	0.002086	1.034891	0.001126
-9.12	0.405634	0.001962	0.525072	0.001048	0.004789	0.002059	1.037272	0.001122
-8.385	0.399907	0.001934	0.524567	0.001047	0.004575	0.002031	1.039719	0.001118
-7.65	0.394165	0.001906	0.52408	0.001046	0.004362	0.002003	1.04223	0.001114

-6.915	0.388408	0.001879	0.523624	0.001045	0.004149	0.001976	1.044842	0.00111
-6.18	0.382633	0.001851	0.523188	0.001044	0.003937	0.001948	1.047528	0.001107
-5.445	0.376833	0.001823	0.522772	0.001044	0.003725	0.00192	1.050289	0.001103
-4.71	0.371013	0.001795	0.522394	0.001048	0.003515	0.001893	1.053174	0.001099
-3.975	0.365178	0.001767	0.522036	0.001051	0.003305	0.001865	1.056131	0.001096
-3.24	0.359331	0.00174	0.521706	0.001054	0.003097	0.001837	1.059177	0.001093
-2.505	0.353467	0.001712	0.521414	0.001058	0.00295	0.00181	1.062335	0.001089
-1.77	0.347585	0.001685	0.521146	0.001062	0.002884	0.001782	1.065575	0.001086
-1.035	0.341686	0.001657	0.520922	0.001066	0.002818	0.001754	1.06894	0.001083
-0.3	0.335769	0.00163	0.520738	0.00107	0.002754	0.001727	1.072412	0.00108
0.435	0.329836	0.001603	0.520584	0.001074	0.00269	0.001699	1.075967	0.001077
1.17	0.323892	0.001575	0.520459	0.001078	0.002626	0.001672	1.079612	0.001074
1.905	0.317932	0.001548	0.520365	0.001082	0.002564	0.001644	1.083348	0.001071
2.64	0.311954	0.001521	0.520299	0.001087	0.002502	0.001616	1.087175	0.001068
3.375	0.305958	0.001494	0.520253	0.001091	0.002482	0.001589	1.091067	0.001065

4.11	0.29994	0.001467	0.52023	0.001096	0.002487	0.001561	1.095067	0.001062
4.845	0.293923	0.00144	0.520219	0.001101	0.002492	0.001533	1.099117	0.001059
5.58	0.287906	0.001413	0.520245	0.001106	0.002498	0.001506	1.1033	0.001057
6.315	0.28188	0.001386	0.520274	0.001112	0.002503	0.001478	1.107541	0.001054
7.05	0.275796	0.00136	0.520335	0.001117	0.002508	0.001451	1.111879	0.001052
7.785	0.269645	0.001334	0.52043	0.001123	0.002514	0.001423	1.116332	0.001049
8.52	0.263473	0.001308	0.520543	0.001129	0.002519	0.001396	1.120929	0.001047
9.255	0.257292	0.001288	0.520697	0.001135	0.002524	0.001374	1.125727	0.001045
9.99	0.251076	0.00128	0.52083	0.001141	0.00253	0.001361	1.130658	0.001042
10.725	0.2448	0.001273	0.521031	0.001148	0.002594	0.001349	1.135805	0.00104
11.46	0.23849	0.001265	0.521281	0.001155	0.002839	0.001336	1.141131	0.001038
12.195	0.232156	0.001258	0.521547	0.001163	0.003085	0.001323	1.146603	0.001036
12.93	0.2258	0.001251	0.521855	0.00117	0.003333	0.001311	1.152343	0.001035
13.665	0.219432	0.001246	0.52218	0.001178	0.003582	0.001299	1.158252	0.001033
14.4	0.213085	0.001247	0.522537	0.001186	0.003833	0.001286	1.164375	0.001031

15.135	0.206687	0.001247	0.522968	0.001195	0.004083	0.001274	1.170812	0.00103
15.87	0.200207	0.001247	0.523371	0.001204	0.004335	0.001262	1.177353	0.001029
16.605	0.193608	0.001248	0.523718	0.001214	0.004586	0.00125	1.183982	0.001027
17.34	0.186606	0.001249	0.524115	0.001223	0.004838	0.001238	1.190877	0.001026
18.075	0.178749	0.00125	0.52458	0.001234	0.005091	0.001226	1.198119	0.001026
18.81	0.170309	0.001251	0.52499	0.001245	0.005343	0.001214	1.205506	0.001025
19.545	0.161689	0.001253	0.525436	0.001256	0.005596	0.001204	1.213417	0.001024
20.28	0.162143	0.001254	0.525941	0.001268	0.005849	0.001196	1.22245	0.001024
21.015	0.162783	0.001257	0.526548	0.001282	0.006102	0.001189	1.232702	0.001023
21.75	0.163438	0.00126	0.527005	0.001297	0.006355	0.001183	1.244574	0.001023
22.485	0.164118	0.001264	0.52735	0.001312	0.006609	0.001178	1.257341	0.001024
23.22	0.16477	0.001267	0.527511	0.001329	0.006863	0.001172	1.270449	0.001025
23.955	0.165409	0.00127	0.527599	0.001347	0.007116	0.001167	1.284479	0.001025
24.69	0.165929	0.001273	0.527951	0.001366	0.00737	0.001162	1.300197	0.001026
25.425	0.166389	0.001272	0.528286	0.001388	0.007624	0.001156	1.316661	0.001029

26.16	0.166783	0.001266	0.52837	0.001412	0.007879	0.001149	1.333693	0.001036
26.895	0.166768	0.001249	0.538816	0.00144	0.008133	0.001135	1.351293	0.001045
27.63	0.165013	0.0012	0.549986	0.001474	0.008387	0.001106	1.36898	0.001054
28.365	0.163143	0.001149	0.564916	0.001511	0.008642	0.001074	1.393024	0.001064
29.1	0.161475	0.0011	0.583296	0.001549	0.008896	0.001044	1.421815	0.001075
29.835	0.160105	0.001066	0.60424	0.001592	0.009149	0.001044	1.455095	0.00109
30	0.159826	0.001069	0.609106	0.001603	0.009206	0.001047	1.468486	0.001094

Implant Stress, Implant Displacement, Cranial stress, Cranial displacement, Scalp Stress, Scalp Displacement, Itanium fixation plate Stress and Itanium fixation plate Displacement variations for the polyetheretherketone plate under Mode 1.

Table S9. Mode 1 stress/displacement, PEEK, skull base excluded.

polyetheretherketone plate				
ICP(mmHg)	Implant Stress (MPa)	Implant Displacement (mm)	Scalp Stress (MPa)	Scalp Displacement (mm)
-15	0.185948	0.001623	0.00651	0.001986
-14.265	0.183554	0.001612	0.006294	0.001946
-13.53	0.18116	0.001601	0.006079	0.001906
-12.795	0.178767	0.00159	0.005863	0.001866
-12.06	0.176376	0.001579	0.005648	0.001826
-11.325	0.173986	0.001568	0.005433	0.001801
-10.59	0.171597	0.001557	0.005218	0.001783
-9.855	0.169209	0.001546	0.005003	0.001766
-9.12	0.166824	0.001535	0.004789	0.00175
-8.385	0.16444	0.001525	0.004575	0.001734

-7.65	0.162057	0.001514	0.004362	0.001718
-6.915	0.159676	0.001503	0.004149	0.001702
-6.18	0.157297	0.001492	0.003937	0.001686
-5.445	0.154921	0.001482	0.003725	0.00167
-4.71	0.152548	0.001471	0.003515	0.001654
-3.975	0.152272	0.001461	0.003305	0.001638
-3.24	0.152313	0.00145	0.003097	0.001622
-2.505	0.15237	0.00144	0.00295	0.001607
-1.77	0.152438	0.00143	0.002884	0.001591
-1.035	0.152525	0.001419	0.002818	0.001576
-0.3	0.152635	0.001409	0.002754	0.00156
0.435	0.15276	0.001399	0.00269	0.001545
1.17	0.152899	0.001389	0.002626	0.00153
1.905	0.153052	0.001378	0.002564	0.001514
2.64	0.153218	0.001368	0.002502	0.001499
3.375	0.153394	0.001359	0.002482	0.001484
4.11	0.153584	0.001349	0.002487	0.001469
4.845	0.153781	0.00134	0.002492	0.001455
5.58	0.153994	0.001331	0.002498	0.00144
6.315	0.154212	0.001322	0.002503	0.001426
7.05	0.154443	0.001314	0.002508	0.001413
7.785	0.154689	0.001305	0.002514	0.0014
8.52	0.15496	0.001297	0.002519	0.001387
9.255	0.155261	0.001288	0.002524	0.001374
9.99	0.155582	0.00128	0.00253	0.001361
10.725	0.155932	0.001273	0.002594	0.001349
11.46	0.156304	0.001265	0.002839	0.001336
12.195	0.156689	0.001258	0.003085	0.001323
12.93	0.157094	0.001251	0.003333	0.001311
13.665	0.157531	0.001246	0.003582	0.001299
14.4	0.157993	0.001247	0.003833	0.001286
15.135	0.158487	0.001247	0.004083	0.001274
15.87	0.158981	0.001247	0.004335	0.001262
16.605	0.159464	0.001248	0.004586	0.00125
17.34	0.159966	0.001249	0.004838	0.001249
18.075	0.160494	0.00125	0.005091	0.00125
18.81	0.161021	0.001251	0.005343	0.001251
19.545	0.161563	0.001253	0.005596	0.001253
20.28	0.162143	0.001254	0.005849	0.001254
21.015	0.162783	0.001257	0.006102	0.001257
21.75	0.163438	0.00126	0.006355	0.00126
22.485	0.164118	0.001264	0.006609	0.001264
23.22	0.16477	0.001267	0.006863	0.001267

23.955	0.165409	0.00127	0.007116	0.00127
24.69	0.165929	0.001273	0.00737	0.001273
25.425	0.166388	0.001272	0.007624	0.001272
26.16	0.166783	0.001266	0.007879	0.001266
26.895	0.166767	0.001249	0.008133	0.001249
27.63	0.165012	0.0012	0.008387	0.0012
28.365	0.163143	0.001149	0.008642	0.001149
29.1	0.161475	0.0011	0.008896	0.0011
29.835	0.160105	0.001066	0.009149	0.001066
30	0.159826	0.001069	0.009206	0.001069

Implant Stress, Implant Displacement, Scalp Stress and Scalp Displacement variations for the polyetheretherketone plate under Mode 1.Stress and displacement of the remaining cranioplasty implant and overlying scalp following resection of the portions adjacent to the anterior skull base. Given that the uneven surface of the implant adjacent to the skull base could lead to aberrant stress and displacement, this region was excluded for subsequent analysis.
